# Ethnic and racial differences in self-reported symptoms, health status, activity level, and missed work at 3 and 6 months following SARS-CoV-2 infection

**DOI:** 10.3389/fpubh.2023.1324636

**Published:** 2024-01-30

**Authors:** Kelli N. O’Laughlin, Robin E. Klabbers, Imtiaz Ebna Mannan, Nicole L. Gentile, Rachel E. Geyer, Zihan Zheng, Huihui Yu, Shu-Xia Li, Kwun C. G. Chan, Erica S. Spatz, Ralph C. Wang, Michelle L’Hommedieu, Robert A. Weinstein, Ian D. Plumb, Michael Gottlieb, Ryan M. Huebinger, Melissa Hagen, Joann G. Elmore, Mandy J. Hill, Morgan Kelly, Samuel McDonald, Kristin L. Rising, Robert M. Rodriguez, Arjun Venkatesh, Ahamed H. Idris, Michelle Santangelo, Katherine Koo, Sharon Saydah, Graham Nichol, Kari A. Stephens, the INSPIRE Group

**Affiliations:** ^1^Department of Emergency Medicine, University of Washington, Seattle, WA, United States; ^2^Department of Global Health, University of Washington, Seattle, WA, United States; ^3^Center for Outcomes Research and Evaluation, Yale School of Medicine, New Haven, CT, United States; ^4^Department of Family Medicine, University of Washington, Seattle, WA, United States; ^5^Department of Laboratory Medicine and Pathology, University of Washington, Seattle, WA, United States; ^6^Post-COVID Rehabilitation and Recovery Clinic, University of Washington, Seattle, WA, United States; ^7^Section of Cardiovascular Medicine, Yale School of Medicine, New Haven, CT, United States; ^8^Department of Biostatistics, University of Washington, Seattle, WA, United States; ^9^Department of Health Systems and Population Health, University of Washington, Seattle, WA, United States; ^10^Department of Epidemiology, Yale School of Public Health, New Haven, CT, United States; ^11^Yale Center for Outcomes Research and Evaluation, Yale School of Medicine, New Haven, CT, United States; ^12^Department of Emergency Medicine, University of California San Francisco, San Francisco, CA, United States; ^13^Division of General Internal Medicine and Health Services Research, David Geffen School of Medicine at UCLA, Los Angeles, CA, United States; ^14^Divisions of Infectious Diseases, Department of Internal Medicine, Rush University Medical Center, Chicago, IL, United States; ^15^Department of Medicine, Cook County Hospital, Chicago, IL, United States; ^16^National Center for Immunizations and Respiratory Diseases, U.S. Centers for Disease Control and Prevention, Atlanta, GA, United States; ^17^Department of Emergency Medicine, Rush University Medical Center, Chicago, IL, United States; ^18^UTHealth Houston McGovern Medical School Department of Emergency Medicine, Houston, TX, United States; ^19^Department of Emergency Medicine, Sidney Kimmel Medical College, Thomas Jefferson University, Philadelphia, PA, United States; ^20^Department of Emergency Medicine, University of Texas Southwestern Medical Center, Dallas, TX, United States; ^21^Clinical Informatics Center, University of Texas Southwestern Medical Center, Dallas, TX, United States; ^22^Center for Connected Care, Thomas Jefferson University, Philadelphia, PA, United States; ^23^Department of Emergency Medicine, Yale School of Medicine, New Haven, CT, United States; ^24^Biomedical Informatics and Medical Education, University of Washington, Seattle, WA, United States

**Keywords:** COVID-19, disparities, cohort, race, ethnicity, SARS-CoV-2, survey

## Abstract

**Introduction:**

Data on ethnic and racial differences in symptoms and health-related impacts following SARS-CoV-2 infection are limited. We aimed to estimate the ethnic and racial differences in symptoms and health-related impacts 3 and 6 months after the first SARS-CoV-2 infection.

**Methods:**

Participants included adults with SARS-CoV-2 infection enrolled in a prospective multicenter US study between 12/11/2020 and 7/4/2022 as the primary cohort of interest, as well as a SARS-CoV-2-negative cohort to account for non-SARS-CoV-2-infection impacts, who completed enrollment and 3-month surveys (*N* = 3,161; 2,402 SARS-CoV-2-positive, 759 SARS-CoV-2-negative). Marginal odds ratios were estimated using GEE logistic regression for individual symptoms, health status, activity level, and missed work 3 and 6 months after COVID-19 illness, comparing each ethnicity or race to the referent group (non-Hispanic or white), adjusting for demographic factors, social determinants of health, substance use, pre-existing health conditions, SARS-CoV-2 infection status, COVID-19 vaccination status, and survey time point, with interactions between ethnicity or race and time point, ethnicity or race and SARS-CoV-2 infection status, and SARS-CoV-2 infection status and time point.

**Results:**

Following SARS-CoV-2 infection, the majority of symptoms were similar over time between ethnic and racial groups. At 3 months, Hispanic participants were more likely than non-Hispanic participants to report fair/poor health (OR: 1.94; 95%CI: 1.36–2.78) and reduced activity (somewhat less, OR: 1.47; 95%CI: 1.06–2.02; much less, OR: 2.23; 95%CI: 1.38–3.61). At 6 months, differences by ethnicity were not present. At 3 months, Other/Multiple race participants were more likely than white participants to report fair/poor health (OR: 1.90; 95% CI: 1.25–2.88), reduced activity (somewhat less, OR: 1.72; 95%CI: 1.21–2.46; much less, OR: 2.08; 95%CI: 1.18–3.65). At 6 months, Asian participants were more likely than white participants to report fair/poor health (OR: 1.88; 95%CI: 1.13–3.12); Black participants reported more missed work (OR, 2.83; 95%CI: 1.60–5.00); and Other/Multiple race participants reported more fair/poor health (OR: 1.83; 95%CI: 1.10–3.05), reduced activity (somewhat less, OR: 1.60; 95%CI: 1.02–2.51; much less, OR: 2.49; 95%CI: 1.40–4.44), and more missed work (OR: 2.25; 95%CI: 1.27–3.98).

**Discussion:**

Awareness of ethnic and racial differences in outcomes following SARS-CoV-2 infection may inform clinical and public health efforts to advance health equity in long-term outcomes.

## Introduction

The COVID-19 pandemic accentuated health disparities. Early in the pandemic, ethnic and racial minoritized (1, 2) populations were reported to be at greater risk of SARS-CoV-2 infection in association with their overrepresentation in the essential workforce (3, 4), fewer opportunities to work from home (4–7), and less ability to practice social distancing (4, 5, 7, 8). Ethnic or racial minoritized individuals with acute SARS-CoV-2 infection had greater barriers to care, including underinsurance (9), lack of primary care (10), and greater economic consequences from missed work (11, 12). Disparities in health outcomes following acute SARS-CoV-2 infection included higher rates of hospitalization and SARS-CoV-2-related mortality among Black and Hispanic populations (13–20). Converging systems of oppression for ethnic and racial minoritized populations magnified health disparities (21, 22).

Disparities in recovery after SARS-CoV-2 infection remain largely under-explored (23–25). Limitations in the few studies that have reported on ethnic and racial differences in recovery from SARS-CoV-2 include heterogeneity in follow-up duration and definition of post-COVID conditions (26–28), inconsistency of findings (29–32), limited focus on symptom presence (as opposed to impact) (29–31), and lack of adjustment for potential confounding by social health determinants (29, 30). We sought to evaluate symptoms and health-related impacts following SARS-CoV-2 infection by ethnicity and race to inform effective and equitable health interventions.

## Methods

### Study design and participant recruitment

This is a secondary analysis of data from the Innovative Support for Patients with SARS-CoV-2 Infections Registry (INSPIRE), a multicenter, longitudinal cohort study of the sequelae of SARS-CoV-2 in the United States. Adult participants were followed prospectively with patient-reported outcomes collected every 3 months via survey and linked to digital health data. Additional methods of the parent study were described previously (33). In this study, we focus on reporting results among SARS-CoV-2-positive participants to address our primary objective, namely to assess for differences in symptoms and health-related impacts by ethnicity and race following a first SARS-CoV-2 infection. The SARS-CoV-2-negative cohort was included in the analysis to account for non-SARS-CoV-2 impacts.

### Study sample

Participants with self-reported symptoms suggestive of acute SARS-CoV-2 infection who tested SARS-CoV-2-positive or SARS-CoV-2-negative within the past 42 days were eligible for enrollment in INSPIRE. Participants who completed enrollment and the 3-month post-enrollment surveys were included in this analysis; among those included in the analysis, 6-month post-enrollment survey data were included as available. Participants with a prior SARS-CoV-2-positive test (>42 days before enrollment) were excluded.

### Exposures

Ethnicity and race were self-reported at enrollment. Ethnicity was reported as Hispanic or non-Hispanic. Race was reported as American Indian or Alaska Native, Asian, Black or African American, Native Hawaiian or Other Pacific Islander, white, and/or ‘some other race’. This approach was in accordance with the U.S. Office of Management and Budget (OMB) standards (34). Ethnicity and race data were collected separately, first asking participants to indicate whether they were of Hispanic, Latin, or Spanish origin and then asking them to select their race category to permit the most granular presentation of findings and because this is the preferred method for data collection (35). For the analysis, due to the small sample size for some subcategories, race data were collapsed into four categories: Asian, Black, Other/Multiple (participants who identified as American Indian/Alaska Native, Native Hawaiian/Other Pacific Islander, ‘some other race’, and who selected two or more races), and white.

### Other variables

Information on demographics, social determinants of health, and substance use was collected at enrollment. Social determinants of health were assessed using a validated tool and included health insurance status, housing insecurity, food insecurity, utility access, transportation access, and employment (36). Substance use in the past 12 months was assessed for tobacco, alcohol, prescription drugs, marijuana, and illicit drugs. Pre-existing health conditions were collected in the 3-month survey and included asthma, hypertension, diabetes, obesity, emphysema or chronic obstructive pulmonary disease, heart conditions, smoking/tobacco consumption, and kidney and liver disease. Acute SARS-CoV-2 infection status was reported at enrollment and confirmed using the electronic health record (EHR) and/or test result image. In the 3- and 6-month surveys, participants were asked about new SARS-CoV-2 infections. Those who were SARS-CoV-2-negative at enrollment and who subsequently reported testing SARS-CoV-2-positive in their 3- or 6-month survey were censored at that time. COVID-19 vaccination status (receiving any COVID-19 vaccine) was assessed through self-report and EHR data.

### Outcomes

The CDC’s Person Under Investigation for SARS-CoV-2 symptom list was used to assess 21 COVID-19-like symptoms and/or “other symptoms” at enrollment, 3 months, and 6 months (37). Health status (5-point scale, excellent to poor), activity level compared to before SARS-CoV-2-like symptoms (same, somewhat less, and much less), and missed work due to health reasons in the past 3 months (0–5 workdays, 6–10 workdays, 10–20 workdays, up to 4 weeks, and do not work; overlap of intervals reflects the answer options presented in the survey) were assessed at 3 and 6 months.

### Statistical methods

We described socio-demographic and clinical characteristics across ethnicity and race groups and displayed frequency counts by initial SARS-CoV-2 test results. Outcomes were described at enrollment, 3 months, and 6 months after SARS-CoV-2 infection for each ethnic and racial group.

We used generalized estimating equations (GEE) logistic regression to model the association between ethnicity and race (separately) and study outcomes (symptoms, health status, activity level, and missed work) at 3 and 6 months. We leveraged data from the full dataset (including those with and without acute SARS-CoV-2) to fit GEE models with robust standard errors. Independent variables include (1) demographic characteristics (age, gender, education, and family income), social determinants of health, tobacco use, substance use, pre-existing health conditions, and COVID-19 vaccination status (vaccinated or not vaccinated before the index SARS-CoV-2 test); and (2) interactions between ethnicity or race and time point, ethnicity or race and SARS-CoV-2 infection status, and SARS-CoV-2 infection status and time point. Time point was modeled as a categorical variable to account for a non-linear trajectory. Social determinants of health were considered a binary variable coded as “any problem” (vs. no problem) if housing, food security, access to utilities, or access to transportation were unstable (Supplementary Material 1). Substance use was included as present if there was a ‘moderate to severe problem’ with at least one substance. Individual comorbidities were included as indicator variables. Where appropriate, variable subgroups were collapsed to create larger subgroups.

Using the GEE models, we calculated marginal odds ratios, for brevity referred to as odds ratios hereafter, of individual symptoms (yes, no) by comparing each ethnicity and racial group to the corresponding referent group (non-Hispanic for ethnicity and white for race). For the outcome ‘health status’, we estimated the odds ratio of being in ‘very good’ or ‘excellent’ health and being in ‘fair’ or ‘poor’ health compared to ‘good’ health. For activity level, the odds ratio of being ‘somewhat less’ and being ‘much less’ able to do activities was estimated compared to being the ‘same as before’. For missed work, the odds ratio of missing >5 workdays in the prior 3 months due to health reasons was compared to ‘0 to 5 days’.

We did not adjust for multiple comparisons, given the exploratory nature of this study. All tests were two-sided with an alpha criterion of 0.05. Statistical analyses were performed using the GENMOD procedure of SAS 9.4 (SAS Institute Inc., Cary, NC). Additional information about our GEE methods is available (Supplementary Material 2).

### Human subjects approval

This study was approved by the Institutional Review Boards of all eight study sites (33).

## Results

**Figure 1:**
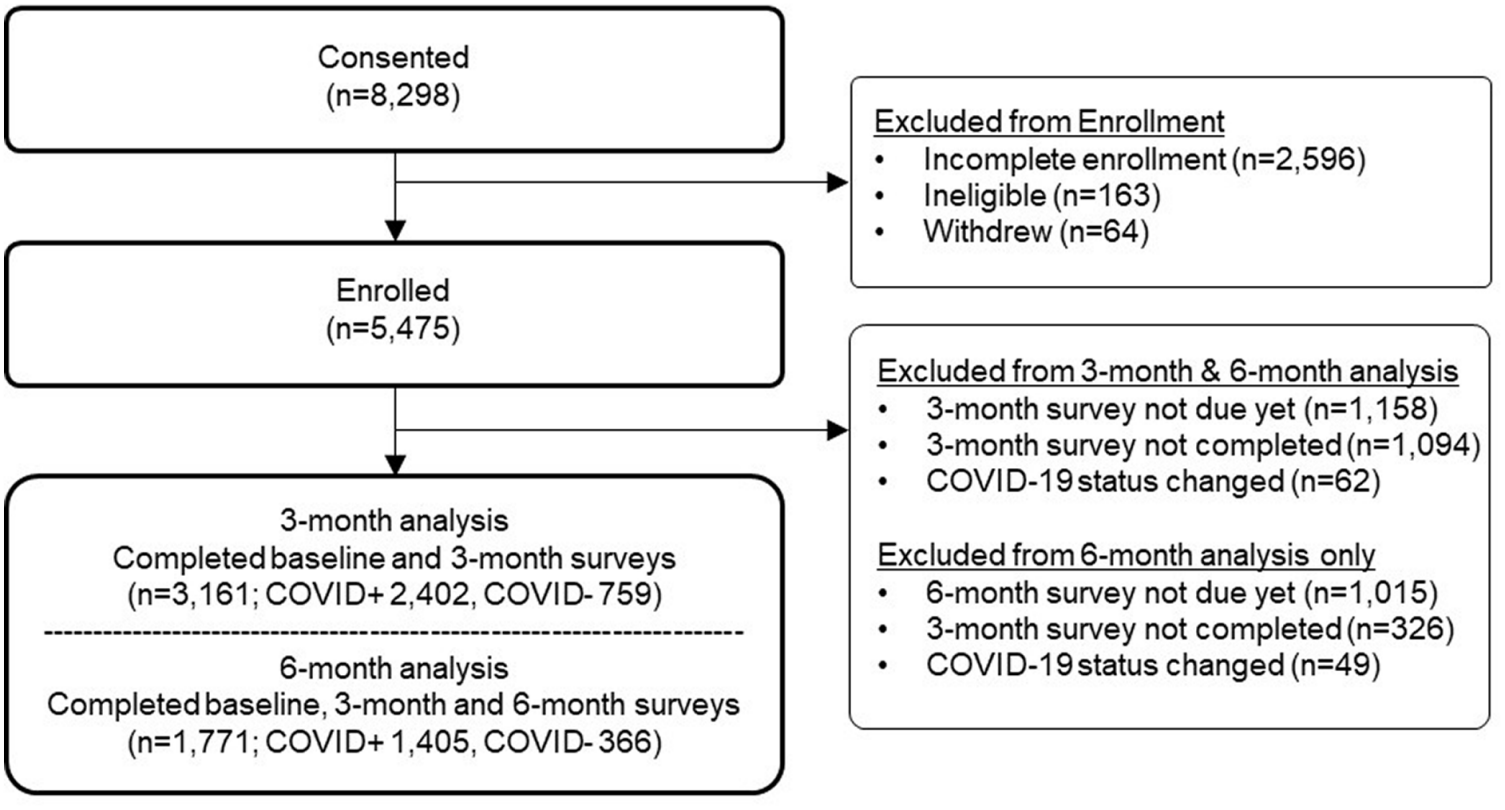
INSPIRE participant flow diagram.

The participant flow diagram is shown in Figure 1. Three-month data were available for all participants (*N* = 3,161), and 6-month data were available for 1,771 participants. Of the 3,161 total participants, ethnicity was reported by 3,155 participants, and race was reported by 3,133 participants. There were differences by ethnicity and race in the proportion of participants completing the follow-up surveys, with lower completion among Hispanic compared to non-Hispanic participants and among Black participants compared to other race groups (Supplementary Material 3).

### Participant characteristics

Among 2,354 SARS-CoV-2-positive participants with ethnicity data, 330 (14.0%) were Hispanic and 2,024 (86.0%) were non-Hispanic. Among 801 SARS-CoV-2-negative participants with ethnicity data, 132 (16.5%) were Hispanic and 669 (83.5%) were non-Hispanic. Assessing both SARS-CoV-2-positive and SARS-CoV-2-negative participants, compared with non-Hispanic participants, Hispanic participants were younger, less educated, more likely never married, and had lower family income (Table 1). Hispanic participants were more likely to lack health insurance, experience housing and/or food insecurity, have limited access to utilities and transportation, and be non-health essential workers working outside the home throughout the pandemic. Hispanic participants were more likely to report prescription abuse and pre-existing obesity and less likely to report hypertension and having no pre-existing health conditions. The proportion of SARS-CoV-2-positive participants was similar among Hispanic and non-Hispanic participants (75.2% vs. 71.4%, respectively, value of *p* = 0.089). Hispanic participants were less likely to be vaccinated against COVID-19, and the two groups utilized different COVID-19 testing sites (Table 1).

**Table 1:** Characteristics by ethnicity of adult INSPIRE participants stratified by SARS-CoV-2 infection status (*N* = 3,155).

	SARS-CoV-2-positive^a^			SARS-CoV-2-negative^a^		
	Hispanic (*N* = 330)	Non-Hispanic (*N* = 2,024)	Total (*N* = 2,354)	Hispanic (*N* = 132)	Non-Hispanic (*N* = 669)	Total (*N* = 801)
	*N* (%)	*N* (%)	*N* (%)	*N* (%)	*N* (%)	*N* (%)
*Age*						
18–34 years	179 (54.2)	810 (40.0)	989 (42.0)	62 (47.0)	304 (45.4)	366 (45.7)
35–49 years	107 (32.4)	638 (31.5)	745 (31.6)	48 (36.4)	176 (26.3)	224 (28.0)
50–64 years	36 (10.9)	405 (20.0)	441 (18.7)	20 (15.2)	128 (19.1)	148 (18.5)
65+ years	8 (2.4)	171 (8.4)	179 (7.6)	2 (1.5)	61 (9.1)	63 (7.9)
*Gender*						
Female	214 (66.0)	1,322 (66.6)	1,536 (66.6)	97 (75.8)	476 (72.7)	573 (73.2)
Male	104 (32.1)	641 (32.3)	745 (32.3)	30 (23.4)	160 (24.4)	190 (24.3)
Transgender/Non-binary	6 (1.9)	21 (1.1)	27 (1.2)	1 (0.8)	19 (2.9)	20 (2.6)
*Education*						
Less than high school	10 (3.2)	14 (0.7)	24 (1.0)	3 (2.5)	6 (0.9)	9 (1.2)
High school graduate	33 (10.7)	120 (6.0)	153 (6.7)	17 (13.9)	62 (9.5)	79 (10.2)
Some college	65 (21.1)	282 (14.2)	347 (15.1)	29 (23.8)	120 (18.4)	149 (19.2)
2-year degree	35 (11.4)	137 (6.9)	172 (7.5)	13 (10.7)	52 (8.0)	65 (8.4)
4-year degree	90 (29.2)	718 (36.1)	808 (35.1)	31 (25.4)	157 (24.0)	188 (24.3)
More than 4 years	75 (24.4)	720 (36.2)	795 (34.6)	29 (23.8)	256 (39.2)	285 (36.8)
*Marital status*						
Married/partner	186 (57.1)	1,176 (58.7)	1,362 (58.4)	58 (45.0)	310 (47.0)	368 (46.6)
Divorced/Widowed/Separated	20 (6.1)	200 (10.0)	220 (9.4)	12 (9.3)	86 (13.0)	98 (12.4)
Never married	120 (36.8)	629 (31.4)	749 (32.1)	59 (45.7)	264 (40.0)	323 (40.9)
*Annual family income*						
<10,000	23 (7.0)	105 (5.2)	128 (5.4)	14 (10.6)	58 (8.7)	72 (9.0)
10,000-34,999	62 (18.8)	197 (9.7)	259 (11.0)	22 (16.7)	76 (11.4)	98 (12.2)
35,000-49,999	53 (16.1)	166 (8.2)	219 (9.3)	26 (19.7)	83 (12.4)	109 (13.6)
50,000-74,999	52 (15.8)	269 (13.3)	321 (13.6)	14 (10.6)	91 (13.6)	105 (13.1)
75,000+	115 (34.8)	1,153 (57.0)	1,268 (53.9)	43 (32.6)	288 (43.0)	331 (41.3)
Prefer not to answer	25 (7.6)	134 (6.6)	159 (6.8)	13 (9.8)	73 (10.9)	86 (10.7)
*Health insurance*						
Private only	224 (67.9)	1,507 (74.5)	1,731 (73.5)	90 (68.2)	453 (67.7)	543 (67.8)
Public only	70 (21.2)	367 (18.1)	437 (18.6)	31 (23.5)	170 (25.4)	201 (25.1)
Private and public	8 (2.4)	80 (4.0)	88 (3.7)	0 (0.0)	27 (4.0)	27 (3.4)
None	28 (8.5)	70 (3.5)	98 (4.2)	11 (8.3)	19 (2.8)	30 (3.7)
*Housing insecurity*						
I have a steady place to live	301 (92.3)	1,922 (95.9)	2,223 (95.4)	112 (86.8)	609 (92.1)	721 (91.3)
I have a place to live today, but I am worried about losing it in the future	21 (6.4)	63 (3.1)	84 (3.6)	14 (10.9)	43 (6.5)	57 (7.2)
I do not have a steady place to live	4 (1.2)	20 (1.0)	24 (1.0)	3 (2.3)	9 (1.4)	12 (1.5)
*Food insecurity*						
Often true	30 (9.2)	78 (3.9)	108 (4.6)	14 (10.9)	43 (6.5)	57 (7.2)
Sometimes true	69 (21.2)	209 (10.4)	278 (11.9)	34 (26.4)	101 (15.3)	135 (17.1)
Never true	227 (69.6)	1,718 (85.7)	1,945 (83.4)	81 (62.8)	516 (78.2)	597 (75.7)
*Utility insecurity*						
Never shut off services	296 (90.5)	1,924 (96.0)	2,220 (95.2)	112 (86.8)	618 (93.5)	730 (92.4)
Threatened to shut off services	29 (8.9)	76 (3.8)	105 (4.5)	16 (12.4)	37 (5.6)	53 (6.7)
Already shut off services	2 (0.6)	4 (0.2)	6 (0.3)	1 (0.8)	6 (0.9)	7 (0.9)
*Transportation access*						
It has kept me from medical appointments or getting medications	13 (3.9)	47 (2.3)	60 (2.5)	13 (9.8)	39 (5.8)	52 (6.5)
It has kept me from non-medical meetings, non-medical appointments, work, or getting things that I need	21 (6.4)	55 (2.7)	76 (3.2)	4 (3.0)	32 (4.8)	36 (4.5)
It has not kept me from getting things I need	296 (89.7)	1,922 (95.0)	2,218 (94.2)	115 (87.1)	598 (89.4)	713 (89.0)
*Employment*						
Employed, essential	149 (45.7)	832 (41.5)	981 (42.1)	52 (40.3)	265 (40.2)	317 (40.2)
Employed, non-essential	121 (37.1)	828 (41.3)	949 (40.7)	48 (37.2)	234 (35.5)	282 (35.7)
Not employed	56 (17.2)	345 (17.2)	401 (17.2)	29 (22.5)	161 (24.4)	190 (24.1)
*Were you employed before the coronavirus outbreak?*						
No	56 (17.2)	345 (17.2)	401 (17.2)	29 (22.5)	161 (24.4)	190 (24.1)
Yes	270 (82.8)	1,661 (82.8)	1,931 (82.8)	100 (77.5)	500 (75.6)	600 (75.9)
*Do you work in a healthcare setting such as a hospital, clinic, or nursing/rehabilitation care facility?*						
No	208 (77.0)	1,240 (74.7)	1,448 (75.0)	73 (73.0)	351 (70.3)	424 (70.8)
Yes	262 (3.0)	421 (25.3)	483 (25.0)	27 (27.0)	148 (29.7)	175 (29.2)
*Are you a non-health essential worker that was asked to work outside the home throughout the pandemic?*						
No	178 (65.7)	1,216 (73.4)	1,394 (72.3)	68 (68.0)	365 (73.3)	433 (72.4)
Yes	93 (34.3)	441 (26.6)	534 (27.7)	32 (32.0)	133 (26.7)	165 (27.6)
*Tobacco use*						
Daily or near daily	12 (3.7)	122 (6.1)	134 (5.7)	12 (9.3)	45 (6.8)	57 (7.2)
Weekly	5 (1.5)	42 (2.1)	47 (2.0)	2 (1.6)	13 (2.0)	15 (1.9)
Monthly	4 (1.2)	32 (1.6)	36 (1.5)	1 (0.8)	8 (1.2)	9 (1.1)
Less than monthly	18 (5.5)	95 (4.7)	113 (4.8)	10 (7.8)	24 (3.6)	34 (4.3)
Not at all	287 (88.0)	1,715 (85.5)	2,002 (85.8)	104 (80.6)	569 (86.3)	673 (85.4)
*Excessive alcohol usage*						
Daily or near daily	2 (0.6)	36 (1.8)	38 (1.6)	2 (1.6)	9 (1.4)	11 (1.4)
Weekly	32 (9.8)	214 (10.7)	246 (10.5)	6 (4.7)	47 (7.1)	53 (6.7)
Monthly	44 (13.5)	265 (13.2)	309 (13.3)	22 (17.1)	66 (10.0)	88 (11.2)
Less than monthly	82 (25.1)	443 (22.1)	525 (22.5)	33 (25.6)	144 (21.9)	177 (22.5)
Not at all	167 (51.1)	1,047 (52.2)	1,214 (52.1)	66 (51.2)	393 (59.6)	459 (58.2)
*Prescription abuse*						
Daily or near daily	9 (2.8)	21 (1.0)	30 (1.3)	4 (3.1)	13 (2.0)	17 (2.2)
Weekly	1 (0.3)	11 (0.5)	12 (0.5)	3 (2.3)	8 (1.2)	11 (1.4)
Monthly	7 (2.1)	13 (0.6)	20 (0.9)	1 (0.8)	10 (1.5)	11 (1.4)
Less than monthly	17 (5.2)	50 (2.5)	67 (2.9)	10 (7.8)	21 (3.2)	31 (3.9)
Not at all	293 (89.6)	1,910 (95.3)	2,203 (94.5)	111 (86.0)	607 (92.1)	718 (91.1)
*Marijuana use*						
Daily or near daily	24 (7.4)	94 (4.7)	118 (5.1)	6 (4.7)	38 (5.8)	44 (5.6)
Weekly	18 (5.5)	108 (5.4)	126 (5.4)	5 (3.9)	25 (3.8)	30 (3.8)
Monthly	13 (4.0)	91 (4.5)	104 (4.5)	6 (4.7)	30 (4.6)	36 (4.6)
Less than monthly	33 (10.2)	236 (11.8)	269 (11.5)	12 (9.3)	68 (10.3)	80 (10.2)
Not at all	237 (72.9)	1,476 (73.6)	1,713 (73.5)	100 (77.5)	497 (75.5)	597 (75.9)
*Drug use*						
Daily or near daily	0 (0.0)	8 (0.4)	8 (0.3)	2 (1.6)	4 (0.6)	6 (0.8)
Weekly	1 (0.3)	5 (0.2)	6 (0.3)	0 (0.0)	1 (0.2)	1 (0.1)
Monthly	0 (0.0)	11 (0.5)	11 (0.5)	0 (0.0)	3 (0.5)	3 (0.4)
Less than monthly	19 (5.8)	106 (5.3)	125 (5.4)	3 (2.3)	23 (3.5)	26 (3.3)
Not at all	306 (93.9)	1,875 (93.5)	2,181 (93.6)	124 (96.1)	628 (95.3)	752 (95.4)
*Pre-existing health conditions* ^b^						
Asthma	36 (11.4)	246 (12.6)	282 (12.4)	21 (16.2)	108 (16.5)	129 (16.4)
Hypertension	34 (10.7)	279 (14.2)	313 (13.7)	15 (11.5)	111 (16.9)	126 (16.1)
Diabetes	18 (5.7)	98 (5.0)	116 (5.1)	7 (5.4)	52 (7.9)	59 (7.5)
Obesity	104 (32.8)	514 (26.2)	618 (27.1)	48 (36.9)	191 (29.2)	239 (30.4)
Emphysema/COPD	1 (0.3)	16 (0.8)	17 (0.7)	3 (2.3)	12 (1.8)	15 (1.9)
Heart conditions	5 (1.6)	50 (2.6)	55 (2.4)	5 (3.8)	27 (4.1)	32 (4.1)
Smoking/tobacco Consumption	7 (2.2)	94 (4.8)	101 (4.4)	11 (8.5)	36 (5.5)	47 (6.0)
Kidney disease	3 (0.9)	27 (1.4)	30 (1.3)	1 (0.8)	14 (2.1)	15 (1.9)
Liver disease	1 (0.3)	15 (0.8)	16 (0.7)	3 (2.3)	9 (1.4)	12 (1.5)
None	37 (11.7)	383 (19.5)	420 (18.4)	17 (13.1)	101 (15.4)	118 (15.0)
I do not know	79 (24.9)	427 (21.8)	506 (22.2)	28 (21.5)	107 (16.3)	135 (17.2)
Prefer not to answer	25 (7.9)	95 (4.8)	120 (5.3)	9 (6.9)	42 (6.4)	51 (6.5)
*COVID-19 vaccination* ^c^						
Yes	175 (65.1)	1,164 (68.4)	1,339 (67.9)	73 (68.2)	438 (78.4)	511 (76.7)
*Testing location*						
At home testing kit	22 (6.7)	164 (8.1)	186 (7.9)	9 (6.8)	63 (9.4)	72 (9.0)
Clinic including urgent care	48 (14.6)	300 (14.9)	348 (14.8)	25 (18.9)	124 (18.5)	149 (18.6)
Emergency department	7 (2.1)	86 (4.3)	93 (4.0)	9 (6.8)	50 (7.5)	59 (7.4)
Hospital	33 (10.0)	176 (8.7)	209 (8.9)	14 (10.6)	68 (10.2)	82 (10.2)
Other	36 (10.9)	130 (6.4)	166 (7.1)	21 (15.9)	89 (13.3)	110 (13.7)
Tent/drive-up testing site	183 (55.6)	1,160 (57.5)	1,343 (57.3)	54 (40.9)	275 (41.1)	329 (41.1)

^a^Table excludes participants with missing ethnicity data for COVID-19-positive (*n* = 48) or COVID-19-negative (*n* = 20). ^b^Pre-existing conditions question only asked on 3-month survey beginning 4-14-2021 and has missing data for COVID-19-positive (*n* = 77) and COVID-19-negative (*n* = 16). ^c^COVID-19 vaccination indicates participants with at least one dose before the index SARS-CoV-2 test; Vaccination initiation information was obtained from linked electronic health record data for 922 (29%) participants, using survey data for 1718 (54%) participants, and was missing for 521 (16%) participants.

Among 2,341 SARS-CoV-2-positive participants with race data, 258 (11.0%) were Asian, 186 (7.9%) were Black, 232 (9.9%) were Other/Multiple races, and 1,665 (71.1%) were white (Table 2). Among 792 SARS-CoV-2-negative participants with race data, 117 (14.8%) were Asian, 104 (13.1%) were Black, 64 (8.1%) were Other/Multiple races, and 507 (64.0%) were white. Assessing both SARS-CoV-2-positive and SARS-CoV-2-negative participants, Black participants had the highest prevalence of low family income, lack of health insurance, housing insecurity, and food insecurity, limited utility access, and limited transportation access, followed by Other/Multiple race participants in most instances. Black participants also had the highest prevalence of pre-existing health conditions. Employment in essential services was highest among Black and Other/Multiple race participants, and healthcare setting employment was highest among Asian and Other/Multiple race participants. Black participants were most likely to be tested for COVID-19 in a hospital setting, and COVID-19 vaccination was lowest among Black and Other/Multiple race participants.

**Table 2:** Characteristics by race of adult INSPIRE participants stratified by SARS-CoV-2 status (*N* = 3,133).

	SARS-CoV-2*-*positive^a^					SARS-CoV-2*-*negative^a^				
	Asian (*N* = 258)	Black (*N* = 186)	Other/Multiple (*N* = 232)	White (*N* = 1,665)	Total (*N* = 2,341)	Asian (*N* = 117)	Black (*N* = 104)	Other/Multiple (*N* = 64)	White (*N* = 507)	Total (*N* = 792)
	*N* (%)	*N* (%)	*N* (%)	*N* (%)	*N* (%)	*N* (%)	*N* (%)	*N* (%)	*N* (%)	*N* (%)
Age										
18–34 years	157 (60.9)	71 (38.2)	114 (49.1)	634 (38.1)	976 (41.7)	77 (65.8)	44 (42.3)	38 (59.4)	204 (40.2)	363 (45.8)
35–49 years	74 (28.7)	63 (33.9)	80 (34.5)	525 (31.5)	742 (31.7)	29 (24.8)	30 (28.8)	16 (25.0)	143 (28.2)	218 (27.5)
50–64 years	19 (7.4)	41 (22.0)	32 (13.8)	345 (20.7)	437 (18.7)	8 (6.8)	23 (22.1)	9 (14.1)	107 (21.1)	147 (18.6)
65+ years	8 (3.1)	11 (5.9)	6 (2.6)	161 (9.7)	186 (7.9)	3 (2.6)	7 (6.7)	1 (1.6)	53 (10.5)	64 (8.1)
*Gender*										
Female	170 (66.9)	138 (74.6)	156 (67.8)	1,062 (65.4)	1,526 (66.6)	76 (66.7)	81 (79.4)	42 (68.9)	364 (73.4)	563 (72.8)
Male	83 (32.7)	46 (24.9)	69 (30.0)	539 (33.2)	737 (32.2)	34 (29.8)	20 (19.6)	19 (31.1)	118 (23.8)	191 (24.7)
Transgender/Non-binary	1 (0.4)	1 (0.5)	5 (2.2)	22 (1.4)	29 (1.3)	4 (3.5)	1 (1.0)	0 (0.0)	14 (2.8)	19 (2.5)
*Education*										
Less than High school	1 (0.4)	5 (2.7)	6 (2.6)	14 (0.9)	26 (1.1)	0 (0.0)	3 (2.9)	0 (0.0)	6 (1.2)	9 (1.2)
High school graduate	14 (5.4)	36 (19.6)	18 (7.9)	86 (5.2)	154 (6.7)	10 (8.8)	20 (19.6)	6 (9.4)	43 (8.6)	79 (10.2)
Some College	20 (7.8)	52 (28.3)	50 (21.9)	221 (13.5)	343 (14.8)	27 (23.7)	26 (25.5)	18 (28.1)	78 (15.7)	149 (19.2)
2-year degree	12 (4.7)	24 (13.0)	25 (11.0)	114 (6.9)	175 (7.6)	8 (7.0)	11 (10.8)	4 (6.3)	41 (8.2)	64 (8.2)
4-year degree	105 (40.9)	36 (19.6)	75 (32.9)	591 (36.0)	807 (34.9)	29 (25.4)	22 (21.6)	16 (25.0)	121 (24.3)	188 (24.2)
More than 4 years	105 (40.9)	31 (16.8)	54 (23.7)	615 (37.5)	805 (34.8)	40 (35.1)	20 (19.6)	20 (31.3)	209 (42.0)	289 (37.1)
*Marital status*										
Married/partner	131 (50.8)	66 (36.3)	106 (46.7)	1,046 (63.4)	1,349 (58.2)	41 (35.0)	30 (29.1)	23 (37.1)	265 (53.2)	359 (46.0)
Divorced/Widowed/Separated	9 (3.5)	24 (13.2)	28 (12.3)	158 (9.6)	219 (9.5)	5 (4.3)	22 (21.4)	9 (14.5)	62 (12.4)	98 (12.6)
Never married	118 (45.7)	92 (50.5)	93 (41.0)	446 (27.0)	749 (32.3)	71 (60.7)	51 (49.5)	30 (48.4)	171 (34.3)	323 (41.4)
*Annual family income*										
<10,000	16 (6.2)	27 (14.5)	22 (9.5)	58 (3.5)	123 (5.3)	15 (12.8)	20 (19.2)	5 (7.8)	30 (5.9)	70 (8.8)
10,000-34,999	20 (7.8)	47 (25.3)	37 (15.9)	154 (9.2)	258 (11.0)	7 (6.0)	29 (27.9)	13 (20.3)	47 (9.3)	96 (12.1)
35,000-49,999	15 (5.8)	31 (16.7)	31 (13.4)	138 (8.3)	215 (9.2)	13 (11.1)	17 (16.3)	13 (20.3)	62 (12.2)	105 (13.3)
50,000-74,999	35 (13.6)	28 (15.1)	32 (13.8)	225 (13.5)	320 (13.7)	16 (13.7)	16 (15.4)	7 (10.9)	65 (12.8)	104 (13.1)
75,000+	148 (57.4)	42 (22.6)	89 (38.4)	987 (59.3)	1,266 (54.1)	46 (39.3)	15 (14.4)	15 (23.4)	255 (50.3)	331 (41.8)
Prefer not to answer	24 (9.3)	11 (5.9)	21 (9.1)	103 (6.2)	159 (6.8)	20 (17.1)	7 (6.7)	11 (17.2)	48 (9.5)	86 (10.9)
*Health insurance*										
Private only	212 (82.2)	92 (49.5)	148 (63.8)	1,263 (75.9)	1715 (73.3)	89 (76.1)	49 (47.1)	45 (70.3)	352 (69.4)	535 (67.6)
Public only	34 (13.2)	76 (40.9)	64 (27.6)	266 (16.0)	440 (18.8)	25 (21.4)	49 (47.1)	14 (21.9)	112 (22.1)	200 (25.3)
Private and public	7 (2.7)	4 (2.2)	4 (1.7)	72 (4.3)	87 (3.7)	2 (1.7)	1 (1.0)	2 (3.1)	22 (4.3)	27 (3.4)
None	5 (1.9)	14 (7.5)	16 (6.9)	64 (3.8)	99 (4.2)	1 (0.9)	5 (4.8)	3 (4.7)	21 (4.1)	30 (3.8)
*Housing insecurity*										
I have a steady place to live	253 (98.1)	159 (87.4)	214 (93.4)	1,588 (96.4)	2,214 (95.6)	108 (92.3)	87 (84.5)	55 (88.7)	464 (93.0)	714 (91.4)
I have a place to live today, but I am worried about losing it in the future	5 (1.9)	17 (9.3)	11 (4.8)	46 (2.8)	79 (3.4)	8 (6.8)	11 (10.7)	6 (9.7)	30 (6.0)	55 (7.0)
I do not have a steady place to live	0 (0.0)	6 (3.3)	4 (1.7)	14 (0.8)	24 (1.0)	1 (0.9)	5 (4.9)	1 (1.6)	5 (1.0)	12 (1.5)
*Food insecurity*										
Often true	5 (1.9)	20 (11.0)	18 (7.9)	63 (3.8)	106 (4.6)	3 (2.6)	23 (22.5)	6 (9.7)	25 (5.0)	57 (7.3)
Sometimes true	29 (11.2)	66 (36.3)	34 (14.9)	145 (8.8)	274 (11.8)	20 (17.1)	27 (26.5)	17 (27.4)	71 (14.2)	135 (17.3)
Never true	224 (86.8)	96 (52.7)	176 (77.2)	1,441 (87.4)	1,937 (83.6)	94 (80.3)	52 (51.0)	39 (62.9)	403 (80.8)	588 (75.4)
*Utility insecurity*										
Never shut off services	256 (99.2)	139 (76.4)	215 (93.9)	1,598 (97.0)	2,208 (95.3)	113 (96.6)	82 (79.6)	55 (88.7)	472 (94.6)	722 (92.4)
Threatened to shut off services	2 (0.8)	41 (22.5)	13 (5.7)	47 (2.9)	103 (4.4)	3 (2.6)	20 (19.4)	6 (9.7)	23 (4.6)	52 (6.7)
Already shut off services	0 (0.0)	2 (1.1)	1 (0.4)	3 (0.2)	6 (0.3)	1 (0.9)	1 (1.0)	1 (1.6)	4 (0.8)	7 (0.9)
*Transportation access*										
It has kept me from medical appointments or getting medications	9 (3.5)	13 (7.0)	13 (5.6)	22 (1.3)	57 (2.4)	9 (7.7)	12 (11.5)	4 (6.3)	27 (5.3)	52 (6.6)
It has kept me from non-medical meetings, non-medical appointments, work, or getting things that I need	14 (5.4)	18 (9.7)	11 (4.7)	33 (2.0)	76 (3.2)	7 (6.0)	11 (10.6)	1 (1.6)	17 (3.4)	36 (4.5)
It has not kept me from getting things I need	235 (91.1)	155 (83.3)	208 (89.7)	1,610 (96.7)	2,208 (94.3)	101 (86.3)	81 (77.9)	59 (92.2)	463 (91.3)	704 (88.9)
*Employment*										
Employed, essential	112 (43.4)	89 (48.9)	98 (42.8)	677 (41.1)	976 (42.1)	40 (34.5)	49 (47.6)	26 (41.9)	197 (39.5)	312 (40.0)
Employed, non-essential	101 (39.1)	59 (32.4)	84 (36.7)	702 (42.6)	946 (40.8)	41 (35.3)	35 (34.0)	18 (29.0)	185 (37.1)	279 (35.8)
Not employed	45 (17.4)	34 (18.7)	47 (20.5)	269 (16.3)	395 (17.0)	35 (30.2)	19 (18.4)	18 (29.0)	117 (23.4)	189 (24.2)
*Were you employed before the coronavirus outbreak?*										
No	45 (17.4)	34 (18.7)	47 (20.5)	269 (16.3)	395 (17.0)	35 (29.9)	19 (18.4)	18 (29.0)	117 (23.4)	189 (24.2)
Yes	213 (82.6)	148 (81.3)	182 (79.5)	1,380 (83.7)	1,923 (83.0)	82 (70.1)	84 (81.6)	44 (71.0)	382 (76.6)	592 (75.8)
*Do you work in a healthcare setting such as a hospital, clinic, or nursing/rehabilitation care facility?*										
No	136 (63.8)	105 (70.9)	145 (79.7)	1,058 (76.7)	1,444 (75.1)	51 (63.0)	62 (73.8)	27 (61.4)	276 (72.3)	416 (70.4)
Yes	77 (36.2)	43 (29.1)	37 (20.3)	322 (23.3)	479 (24.9)	30 (37.0)	22 (26.2)	17 (38.6)	106 (27.7)	175 (29.6)
*Are you a non-health essential worker who was asked to work outside the home throughout the pandemic?*										
No	173 (81.2)	99 (66.9)	118 (64.8)	997 (72.4)	1,387 (72.2)	67 (82.7)	53 (63.1)	31 (70.5)	281 (73.8)	432 (73.2)
Yes	40 (18.8)	49 (33.1)	64 (35.2)	380 (27.6)	533 (27.8)	14 (17.3)	31 (36.9)	13 (29.5)	100 (26.2)	158 (26.8)
*Tobacco use*										
Daily or near daily	8 (3.1)	15 (8.2)	17 (7.5)	93 (5.6)	133 (5.7)	4 (3.4)	10 (9.7)	6 (9.7)	36 (7.2)	56 (7.2)
Weekly	4 (1.6)	7 (3.8)	5 (2.2)	32 (1.9)	48 (2.1)	3 (2.6)	1 (1.0)	3 (4.8)	8 (1.6)	15 (1.9)
Monthly	3 (1.2)	1 (0.5)	6 (2.6)	26 (1.6)	36 (1.6)	3 (2.6)	2 (1.9)	2 (3.2)	2 (0.4)	9 (1.2)
Less than monthly	10 (3.9)	12 (6.6)	14 (6.1)	77 (4.7)	113 (4.9)	5 (4.3)	4 (3.9)	4 (6.5)	22 (4.4)	35 (4.5)
Not at all	233 (90.3)	147 (80.8)	186 (81.6)	1,422 (86.2)	1,988 (85.8)	101 (87.1)	86 (83.5)	47 (75.8)	430 (86.3)	664 (85.2)
*Excessive alcohol usage*										
Daily or near daily	2 (0.8)	5 (2.7)	2 (0.9)	30 (1.8)	39 (1.7)	1 (0.9)	3 (2.9)	1 (1.6)	6 (1.2)	11 (1.4)
Weekly	22 (8.5)	19 (10.4)	25 (10.9)	179 (10.9)	245 (10.6)	4 (3.4)	12 (11.7)	2 (3.2)	34 (6.8)	52 (6.7)
Monthly	27 (10.5)	14 (7.7)	28 (12.2)	239 (14.5)	308 (13.3)	16 (13.8)	9 (8.7)	9 (14.5)	50 (10.0)	84 (10.8)
Less than monthly	59 (22.9)	35 (19.2)	59 (25.8)	369 (22.4)	522 (22.5)	26 (22.4)	23 (22.3)	23 (37.1)	100 (20.1)	172 (22.1)
Not at all	148 (57.4)	109 (59.9)	115 (50.2)	832 (50.5)	1,204 (51.9)	69 (59.5)	56 (54.4)	27 (43.5)	308 (61.8)	460 (59.1)
*Prescription abuse*										
Daily or near daily	4 (1.6)	4 (2.2)	7 (3.1)	13 (0.8)	28 (1.2)	2 (1.7)	5 (4.9)	3 (4.8)	7 (1.4)	17 (2.2)
Weekly		0 (0.0)	3 (1.3)	9 (0.5)	12 (0.5)	0 (0.0)	1 (1.0)	2 (3.2)	8 (1.6)	11 (1.4)
Monthly	2 (0.8)	3 (1.6)	6 (2.6)	8 (0.5)	19 (0.8)	2 (1.7)	2 (1.9)	2 (3.2)	5 (1.0)	11 (1.4)
Less than monthly	12 (4.7)	8 (4.4)	8 (3.5)	39 (2.4)	67 (2.9)	3 (2.6)	4 (3.9)	5 (8.1)	16 (3.2)	28 (3.6)
Not at all	240 (93.0)	167 (91.8)	205 (89.5)	1,580 (95.8)	2,192 (94.6)	109 (94.0)	91 (88.3)	50 (80.6)	462 (92.8)	712 (91.4)
*Marijuana use*										
Daily or near daily	4 (1.6)	15 (8.2)	16 (7.0)	80 (4.9)	115 (5.0)	4 (3.4)	8 (7.8)	6 (9.7)	26 (5.2)	44 (5.7)
Weekly	13 (5.0)	11 (6.0)	19 (8.3)	82 (5.0)	125 (5.4)	1 (0.9)	7 (6.9)	2 (3.2)	20 (4.0)	30 (3.9)
Monthly	5 (1.9)	3 (1.6)	13 (5.7)	82 (5.0)	103 (4.4)	9 (7.8)	2 (2.0)	5 (8.1)	19 (3.8)	35 (4.5)
Less than monthly	30 (11.6)	16 (8.8)	35 (15.3)	184 (11.2)	265 (11.4)	12 (10.3)	15 (14.7)	7 (11.3)	46 (9.2)	80 (10.3)
Not at all	206 (79.8)	137 (75.3)	146 (63.8)	1,219 (74.0)	1708 (73.7)	90 (77.6)	70 (68.6)	42 (67.7)	387 (77.7)	589 (75.7)
*Drug use*										
Daily or near daily	0 (0.0)	0 (0.0)	1 (0.4)	7 (0.4)	8 (0.3)	1 (0.9)	2 (1.9)	1 (1.6)	2 (0.4)	6 (0.8)
Weekly	0 (0.0)	1 (0.5)	1 (0.4)	4 (0.2)	6 (0.3)	0 (0.0)	0 (0.0)	0 (0.0)	1 (0.2)	1 (0.1)
Monthly	0 (0.0)	1 (0.5)	5 (2.2)	5 (0.3)	11 (0.5)	0 (0.0)	0 (0.0)	1 (1.6)	2 (0.4)	3 (0.4)
Less than monthly	21 (8.1)	5 (2.7)	11 (4.8)	86 (5.2)	123 (5.3)	1 (0.9)	3 (2.9)	4 (6.5)	18 (3.6)	26 (3.3)
Not at all	237 (91.9)	175 (96.2)	211 (92.1)	1,546 (93.8)	2,169 (93.6)	114 (98.3)	98 (95.1)	56 (90.3)	475 (95.4)	743 (95.4)
*Pre-existing health conditions* ^b^										
Asthma	28 (11.2)	38 (22.0)	42 (18.8)	171 (10.6)	279 (12.3)	13 (11.3)	21 (21.0)	13 (21.3)	82 (16.4)	129 (16.6)
Hypertension	19 (7.6)	47 (27.2)	128 (2.6)	221 (13.7)	315 (13.9)	9 (7.8)	26 (26.0)	8 (13.1)	84 (16.8)	127 (16.4)
Diabetes	10 (4.0)	17 (9.8)	15 (6.7)	74 (4.6)	116 (5.1)	4 (3.5)	16 (16.0)	5 (8.2)	34 (6.8)	59 (7.6)
Obesity	35 (13.9)	65 (37.6)	76 (34.1)	441 (27.3)	617 (27.3)	17 (14.8)	36 (36.0)	19 (31.1)	163 (32.6)	235 (30.3)
Emphysema/COPD	0 (0.0)	4 (2.3)	0 (0.0)	14 (0.9)	18 (0.8)	0 (0.0)	5 (5.0)	1 (1.6)	9 (1.8)	15 (1.9)
Heart Conditions	4 (1.6)	9 (5.2)	5 (2.2)	37 (2.3)	55 (2.4)	2 (1.7)	7 (7.0)	2 (3.3)	22 (4.4)	33 (4.3)
Smoking/tobacco Consumption	7 (2.8)	17 (9.8)	15 (6.7)	62 (3.8)	101 (4.5)	5 (4.3)	6 (6.0)	8 (13.1)	28 (5.6)	47 (6.1)
Kidney Disease	4 (1.6)	6 (3.5)	2 (0.9)	19 (1.2)	31 (1.4)	2 (1.7)	3 (3.0)	1 (1.6)	9 (1.8)	15 (1.9)
Liver Disease	1 (0.4)	3 (1.7)	0 (0.0)	12 (0.7)	16 (0.7)	0 (0.0)	2 (2.0)	0 (0.0)	9 (1.8)	11 (1.4)
None	50 (19.9)	17 (9.8)	33 (14.8)	321 (19.9)	421 (18.6)	27 (23.5)	9 (9.0)	12 (19.7)	70 (14.0)	118 (15.2)
I do not know	84 (33.5)	19 (11.0)	45 (20.2)	351 (21.7)	499 (22.1)	30 (26.1)	14 (14.0)	12 (19.7)	81 (16.2)	137 (17.7)
Prefer not to answer	15 (6.0)	11 (6.4)	16 (7.2)	75 (4.6)	117 (5.2)	13 (11.3)	7 (7.0)	1 (1.6)	27 (5.4)	48 (6.2)
*COVID-19 vaccination* ^**c**^										
Yes	165 (80.5)	72 (46.5)	133 (67.9)	964 (68.6)	1,334 (68.0)	76 (80.9)	61 (73.5)	35 (66.0)	339 (78.5)	511 (77.2)
No	40 (19.5)	83 (53.5)	63 (32.1)	441 (31.4)	627 (32.0)	18 (19.1)	22 (26.5)	18 (34.0)	93 (21.5)	151 (22.8)
*Testing location*										
At home testing kit	24 (9.3)	2 (1.1)	15 (6.5)	144 (8.7)	185 (7.9)	12 (10.3)	7 (6.7)	8 (12.5)	46 (9.1)	73 (9.2)
Clinic including urgent care	21 (8.1)	26 (14.1)	28 (12.1)	271 (16.4)	346 (14.8)	14 (12.0)	16 (15.4)	8 (12.5)	111 (21.9)	149 (18.8)
Emergency department	6 (2.3)	29 (15.7)	11 (4.7)	49 (3.0)	95 (4.1)	7 (6.0)	18 (17.3)	5 (7.8)	26 (5.1)	56 (7.1)
Hospital	18 (7.0)	41 (22.2)	23 (9.9)	128 (7.7)	210 (9.0)	8 (6.8)	17 (16.3)	7 (10.9)	47 (9.3)	79 (10.0)
Other	16 (6.2)	9 (4.9)	23 (9.9)	117 (7.1)	165 (7.1)	27 (23.1)	8 (7.7)	15 (23.4)	59 (11.6)	109 (13.8)
Tent/drive up testing site	173 (67.1)	78 (42.2)	132 (56.9)	948 (57.2)	1,331 (57.1)	49 (41.9)	38 (36.5)	21 (32.8)	218 (43.0)	326 (41.2)

^a^Table excludes participants with missing race data for COVID-19-positive (61) or COVID-19-negative (*n* = 29). ^b^Pre-existing conditions question only asked on 3-month survey beginning 4-14-2021 and has missing data for COVID-19-positive (*n* = 78) and COVID-19-negative (*n* = 16). ^c^COVID-19 vaccination indicates participants with at least one dose prior to the index SARS-CoV-2 test; Vaccination initiation information was obtained from linked electronic health record data for 922 (29%) participants, using survey data for 1718 (54%) participants, and was missing for 521 (16%) participants.

### Symptoms, health status, activity level, and work among SARS-CoV-2-positive participants

At each time point, the reported prevalence of symptoms, health status, activity level, and missed work among SARS-CoV-2-positive participants varied by ethnicity and race (Table 3; results for the SARS-CoV-2-negative cohort included in Supplementary Material 4).

**Table 3:** Symptoms, health status, activity level, and missed work over time among adult INSPIRE SARS-CoV-2-positive participants by ethnicity and race.

	Enrollment^a^						3-Month^a^						6-Month^b^					
	Ethnicity		Race				Ethnicity		Race				Ethnicity		Race			
	Hispanic (*N* = 330)	Non-Hispanic (*N* = 2,024)	Asian (*N* = 258)	Black (*N* = 186)	Other/ Multiple (*N* = 232)	White (*N* = 1,665)	Hispanic (*N* = 330)	Non-Hispanic (*N* = 2,024)	Asian (*N* = 258)	Black (*N* = 186)	Other/ Multiple (*N* = 232)	White (*N* = 1,665)	Hispanic (*N* = 174)	Non-Hispanic (*N* = 1,208)	Asian (*N* = 142)	Black (*N* = 109)	Other/ Multiple (*N* = 124)	White (*N* = 1,000)
Symptom category	*N* (%)	*N* (%)	*N* (%)	*N* (%)	*N* (%)	*N* (%)	*N* (%)	*N* (%)	*N* (%)	*N* (%)	*N* (%)	*N* (%)	*N* (%)	*N* (%)	N (%)	*N* (%)	*N* (%)	*N* (%)
*Constitutional*																		
Tired	257 (78.4)	1,641 (81.7)	204 (79.1)	127 (69.8)	196 (85.6)	1,365 (82.6)	69 (20.9)	465 (23.1)	43 (16.9)	44 (23.8)	69 (29.9)	379 (22.9)	32 (18.7)	241 (20.1)	22 (15.7)	20 (18.7)	30 (24.4)	198 (19.9)
Chills	173 (52.7)	1,013 (50.4)	138 (53.5)	100 (54.9)	126 (55.0)	809 (48.9)	31 (9.4)	115 (5.7)	11 (4.3)	20 (10.8)	18 (7.8)	97 (5.9)	8 (4.7)	54 (4.5)	6 (4.3)	5 (4.7)	3 (2.4)	47 (4.7)
Feeling hot	159 (48.5)	941 (46.9)	113 (43.8)	81 (44.5)	130 (56.8)	767 (46.4)	22 (6.7)	106 (5.3)	11 (4.3)	14 (7.6)	19 (8.2)	85 (5.1)	7 (4.1)	53 (4.4)	8 (5.7)	4 (3.7)	3 (2.4)	46 (4.6)
Fever	105 (32.0)	613 (30.5)	90 (34.9)	54 (29.7)	66 (28.8)	505 (30.6)	15 (4.5)	61 (3.0)	6 (2.4)	9 (4.9)	11 (4.8)	51 (3.1)	2 (1.2)	27 (2.3)	4 (2.9)	3 (2.8)	2 (1.6)	19 (1.9)
Shakes	65 (19.8)	288 (14.3)	32 (12.4)	43 (23.6)	49 (21.4)	226 (13.7)	13 (3.9)	40 (2.0)	7 (2.7)	9 (4.9)	5 (2.2)	32 (1.9)	2 (1.2)	17 (1.4)	4 (2.9)	1 (0.9)	1 (0.8)	12 (1.2)
*HEENT* ^c^																		
Headache	217 (66.2)	1,334 (66.4)	165 (64.0)	114 (62.6)	153 (66.8)	1,109 (67.1)	67 (20.3)	265 (13.2)	25 (9.8)	32 (17.3)	44 (19.0)	235 (14.2)	27 (15.8)	131 (10.9)	13 (9.3)	16 (15.0)	15 (12.2)	115 (11.6)
Runny nose	196 (59.8)	1,407 (70.1)	180 (69.8)	93 (51.1)	148 (64.6)	1,174 (71.0)	36 (10.9)	193 (9.6)	20 (7.8)	20 (10.8)	28 (12.1)	160 (9.7)	11 (6.4)	114 (9.5)	13 (9.3)	9 (8.4)	12 (9.8)	91 (9.2)
Loss of smell	147 (44.8)	975 (48.6)	117 (45.3)	80 (44.0)	116 (50.7)	798 (48.3)	45 (13.6)	267 (13.3)	26 (10.2)	22 (11.9)	30 (13.0)	232 (14.0)	13 (7.6)	126 (10.5)	15 (10.7)	8 (7.5)	7 (5.7)	108 (10.9)
Loss of taste	133 (40.5)	895 (44.6)	103 (39.9)	75 (41.2)	111 (48.5)	733 (44.3)	36 (10.9)	215 (10.7)	22 (8.6)	21 (11.4)	27 (11.7)	177 (10.7)	7 (4.1)	100 (8.3)	8 (5.7)	7 (6.5)	2 (1.6)	91 (9.2)
Sore throat	180 (54.9)	1,142 (56.9)	188 (72.9)	80 (44.0)	123 (53.7)	927 (56.1)	33 (10.0)	149 (7.4)	26 (10.2)	16 (8.6)	27 (11.7)	114 (6.9)	11 (6.4)	74 (6.2)	9 (6.4)	6 (5.6)	7 (5.7)	63 (6.3)
Loss of hair	20 (6.1)	70 (3.5)	9 (3.5)	8 (4.4)	9 (3.9)	61 (3.7)	34 (10.3)	114 (5.7)	15 (5.9)	15 (8.1)	16 (6.9)	99 (6.0)	14 (8.2)	68 (5.7)	7 (5.0)	8 (7.5)	9 (7.3)	56 (5.6)
*Pulmonary*																		
Cough	188 (57.3)	1,266 (63.0)	165 (64.0)	96 (52.7)	135 (59.0)	1,053 (63.7)	25 (7.6)	145 (7.2)	22 (8.6)	20 (10.8)	23 (10.0)	109 (6.6)	7 (4.1)	64 (5.3)	9 (6.4)	6 (5.6)	5 (4.1)	52 (5.2)
Shortness of breath	110 (33.5)	591 (29.4)	56 (21.7)	69 (37.9)	84 (36.7)	486 (29.4)	34 (10.3)	182 (9.0)	6 (2.4)	23 (12.4)	36 (15.6)	150 (9.0)	16 (9.4)	89 (7.4)	5 (3.6)	10 (9.3)	14 (11.4)	75 (7.5)
Wheezing	37 (11.3)	217 (10.8)	20 (7.8)	26 (14.3)	39 (17.0)	168 (10.2)	8 (2.4)	54 (2.7)	5 (2.0)	4 (2.2)	15 (6.5)	39 (2.4)	5 (2.9)	29 (2.4)	1 (0.7)	3 (2.8)	6 (4.9)	23 (2.3)
*Cardiovascular*																		
Chest pains	79 (24.1)	487 (24.3)	40 (15.5)	55 (30.2)	68 (29.7)	400 (24.2)	20 (6.1)	105 (5.2)	9 (3.5)	12 (6.5)	15 (6.5)	88 (5.3)	6 (3.5)	49 (4.1)	6 (4.3)	5 (4.7)	6 (4.9)	41 (4.1)
Palpitations	33 (10.1)	158 (7.9)	12 (4.7)	12 (6.6)	28 (12.2)	138 (8.3)	18 (5.5)	69 (3.4)	6 (2.4)	9 (4.9)	11 (4.8)	61 (3.7)	11 (6.4)	38 (3.2)	1 (0.7)	4 (3.7)	8 (6.5)	34 (3.4)
*Gastrointestinal*																		
Diarrhea	88 (26.8)	429 (21.4)	44 (17.1)	54 (29.7)	65 (28.4)	353 (21.4)	17 (5.2)	60 (3.0)	5 (2.0)	6 (3.2)	7 (3.0)	60 (3.6)	5 (2.9)	32 (2.7)	5 (3.6)	2 (1.9)	3 (2.4)	28 (2.8)
Nausea or vomiting	79 (24.1)	347 (17.3)	36 (14.0)	42 (23.1)	59 (25.8)	292 (17.7)	13 (3.9)	59 (2.9)	7 (2.7)	6 (3.2)	9 (3.9)	51 (3.1)	8 (4.7)	34 (2.8)	2 (1.4)	5 (4.7)	4 (3.3)	31 (3.1)
Abdominal pain	44 (13.4)	214 (10.7)	20 (7.8)	28 (15.4)	38 (16.6)	171 (10.3)	10 (3.0)	43 (2.1)	5 (2.0)	4 (2.2)	6 (2.6)	39 (2.4)	12 (7.0)	31 (2.6)	1 (0.7)	2 (1.9)	4 (3.3)	35 (3.5)
*Musculoskeletal*																		
Aches	189 (57.6)	1,163 (57.9)	155 (60.1)	107 (58.8)	140 (61.1)	938 (56.7)	48 (14.5)	249 (12.4)	20 (7.8)	33 (17.8)	43 (18.6)	200 (12.1)	22 (12.9)	139 (11.6)	13 (9.3)	18 (16.8)	14 (11.4)	116 (11.7)
Joint pains	106 (32.3)	567 (28.2)	51 (19.8)	55 (30.2)	73 (31.9)	486 (29.4)	40 (12.1)	221 (11.0)	23 (9.0)	32 (17.3)	32 (13.9)	172 (10.4)	15 (8.8)	131 (10.9)	5 (3.6)	15 (14.0)	15 (12.2)	112 (11.3)
*Symptom summary*																		
Other symptoms	42 (12.8)	281 (14.0)	21 (8.1)	15 (8.2)	44 (19.2)	242 (14.6)	13 (3.9)	91 (4.5)	6 (2.4)	4 (2.2)	14 (6.1)	82 (4.9)	10 (5.8)	49 (4.1)	3 (2.1)	3 (2.8)	4 (3.3)	50 (5.0)
≥3 symptoms (not including others)	293 (89.3)	1845 (91.9)	241 (93.4)	150 (82.4)	204 (89.1)	1,528 (92.4)	82 (24.8)	430 (21.4)	39 (15.3)	54 (29.2)	63 (27.3)	341 (20.6)	38 (22.2)	225 (18.8)	19 (13.6)	27 (25.2)	26 (21.1)	192 (19.3)
No symptoms	7 (2.1)	26 (1.3)	0 (0.0)	6 (3.3)	5 (2.2)	22 (1.3)	172 (52.1)	1,148 (57.1)	165 (64.7)	99 (53.5)	115 (49.8)	929 (56.0)	97 (56.7)	714 (59.5)	90 (64.3)	62 (57.9)	64 (52.0)	590 (59.4)
*Health status*																		
Excellent							26 (8.0)	212 (10.6)	29 (11.4)	20 (10.9)	21 (9.2)	167 (10.2)	17 (9.9)	137 (11.4)	17 (12.1)	10 (9.3)	11 (8.9)	113 (11.4)
Very good							78 (24.0)	559 (28.0)	56 (22.0)	31 (16.8)	47 (20.6)	500 (30.4)	49 (28.5)	354 (29.4)	43 (30.5)	21 (19.4)	32 (25.8)	310 (31.2)
Good							102 (31.4)	746 (37.3)	103 (40.6)	67 (36.4)	68 (29.8)	607 (36.9)	54 (31.4)	399 (33.2)	37 (26.2)	37 (34.3)	33 (26.6)	340 (34.2)
Fair							86 (26.5)	380 (19.0)	57 (22.4)	47 (25.5)	66 (28.9)	290 (17.6)	33 (19.2)	257 (21.4)	40 (28.4)	27 (25.0)	34 (27.4)	187 (18.8)
Poor							29 (8.9)	92 (4.6)	8 (3.1)	17 (9.2)	23 (10.1)	73 (4.4)	17 (9.9)	46 (3.8)	4 (2.9)	8 (7.4)	12 (9.7)	39 (3.9)
Do not know							4 (1.2)	7 (0.3)	1 (0.4)	0 (0.0)	2 (0.9)	8 (0.5)	2 (1.2)	9 (0.7)	0 (0.0)	4 (3.7)	2 (1.6)	6 (0.6)
No answer							0 (0.0)	3 (0.1)	0 (0.0)	2 (1.1)	1 (0.4)	0 (0.0)	0 (0.0)	1 (0.1)	0 (0.0)	1 (0.9)	0 (0.0)	0 (0.0)
*Activity level^d^*																		
Same as before							191 (58.8)	1,349 (67.5)	192 (75.6)	100 (54.3)	125 (54.8)	1,114 (67.8)	109 (63.4)	822 (68.3)	105 (74.5)	64 (59.3)	74 (59.7)	678 (68.1)
Somewhat less than before							99 (30.5)	502 (25.1)	53 (20.9)	61 (33.2)	76 (33.3)	404 (24.6)	45 (26.2)	286 (23.8)	32 (22.7)	25.9 (28)	34 (27.4)	239 (24.0)
Much less than before							35 (10.8)	147 (7.4)	9 (3.5)	23 (12.5)	27 (11.8)	126 (7.7)	18 (10.5)	95 (7.9)	4 (2.8)	14.8 (16)	16 (12.9)	78 (7.8)
*Missed work due to health reasons past 3 months*																		
0–5 workdays							213 (65.5)	1,376 (68.9)	198 (78.0)	99 (53.8)	140 (61.4)	1,147 (69.8)	119 (69.2)	889 (73.9)	114 (80.9)	60 (55.6)	78 (62.9)	752 (75.6)
6–10 workdays							30 (9.2)	156 (7.8)	12 (4.7)	26 (14.1)	19 (8.3)	125 (7.6)	11 (6.4)	70 (5.8)	8 (5.7)	12 (11.1)	10 (8.1)	50 (5.0)
11–20 workdays							16 (4.9)	79 (4.0)	7 (2.8)	10 (5.4)	12 (5.3)	65 (4.0)	9 (5.2)	34 (2.8)	2 (1.4)	10 (9.3)	9 (7.3)	20 (2.0)
Up to 4 weeks							16 (4.9)	58 (2.9)	9 (3.5)	16 (8.7)	15 (6.6)	34 (2.1)	6 (3.5)	23 (1.9)	3 (2.1)	5 (4.6)	5 (4.0)	15 (1.5)
Do not work							50 (15.4)	328 (16.4)	28 (11.0)	33 (17.9)	42 (18.4)	272 (16.6)	27 (15.7)	187 (15.5)	14 (9.9)	21 (19.4)	22 (17.7)	158 (15.9)

^a^Included COVID-19-positive participants who completed both enrollment and 3-month surveys; excluded participants with missing ethnicity (*n* = 48) and with missing symptom responses (*n* = 18 for enrollment survey; *n* = 12 for 3-month survey); excluded participants with missing race (*n* = 61) and missing symptom responses (*n* = 19 for enrollment survey; *n* = 12 for 3-month survey). ^b^Included COVID-19-positive participants who completed enrollment, 3-month, and 6-month surveys; excluded participants with missing ethnicity (*n* = 23) and with missing symptom responses (*n* = 11); excluded participants with missing race (*n* = 30) and with missing symptom responses (*n* = 11). ^c^HEENT = head, ears, eyes, nose, throat. ^d^Excluded participants with missing health status (*n* = 30 for 3-month survey; *n* = 7 for 6-month survey), or activity level (*n* = 31 for 3-month survey; *n* = 7 for 6-month survey), or missed work variable (*n* = 32 for 3-month survey; *n* = 7 for 6-month survey).

### Association between ethnicity and race and study outcomes among SARS-CoV-2-positive participants

**Figure 2:**
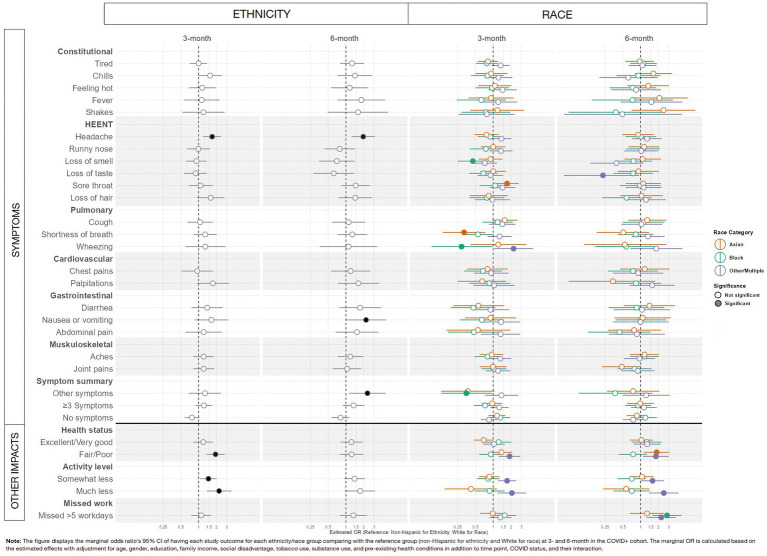
Forest plots to demonstrate study outcomes by ethnicity and race using adjusted analyses among adult SARS-CoV-2-positive INSPIRE participants.

Figure 2 presents adjusted odds ratios for having 21 COVID-like symptoms or “other symptoms” (22 symptoms queried total) comparing Hispanic to non-Hispanic participants and comparing different races to white participants who tested positive for SARS-CoV-2 at 3 and 6 months (entire cohort results are included in Supplementary Materials 5.1–5.3 GEE adjusted odds ratio output).

Compared to non-Hispanic participants, at 3 and 6 months, Hispanic participants reported headaches more frequently (3 months, OR: 1.70, 95%CI: 1.20–2.42; 6 months, OR: 1.97, 95%CI: 1.25–3.11), and at 6 months, they reported more nausea or vomiting (OR: 2.20, 95%CI: 1.01–4.77) and ‘other symptoms’ (OR: 2.32, 95%CI: 1.14–4.72) (Figure 2; Supplementary Material 5.1).

By race, compared to white participants, at 3 months, Asian participants reported more sore throat (OR: 1.72, 95%CI: 1.09–2.72) and less shortness of breath (OR: 0.32, 95%CI: 0.14–0.74); Black participants reported less loss of smell (OR: 0.45, 95%CI: 0.25–0.82), wheezing (OR: 0.29, 95%CI: 0.09–0.98) and ‘other symptoms’ (OR: 0.35, 95%CI: 0.13–0.95); and Other/Multiple race participants reported more wheezing (OR: 2.22, 95%CI: 1.04–4.75). At 6 months, Other/Multiple race participants had less loss of smell than white participants (OR: 0.23, 95%CI: 0.05–0.95) (Figure 2; Supplementary Materials 5.2, 5.3).

Evaluating health status, activity level, and missed work at 3 and 6 months after SARS-CoV-2 infection, some minoritized groups had worse health status, less physical activity, and more missed days of work. At 3 months, Hispanic compared to non-Hispanic participants were more likely to report fair/poor health (OR: 1.94, 95%CI: 1.36–2.78) and less activity (somewhat less, OR: 1.47, 95%CI: 1.06–2.02; much less, OR: 2.23, 95%CI: 1.38–3.61) (Figure 2, estimates reported in Supplementary Material 5.1). These differences in health and activity level by ethnicity were not found at 6 months (Figure 2, estimates reported in Supplementary Material 5.1). There were no significant differences in missed work at 3 or 6 months by ethnicity.

By race, there were differences in health status and activity level at 3 months and in health status, activity level, and work missed at 6 months. At 3 months, Other/Multiple race compared to white participants fared worse in terms of health (fair/poor health, OR: 1.90, 95%CI: 1.25–2.88) and activity level (somewhat less, OR: 1.72, 95%CI: 1.21–2.46; much less, OR: 2.08, 95%CI: 1.18–3.65). At 6 months, these differences persisted for Other/Multiple race participants in terms of health (fair/poor health, OR: 1.83, 95%CI: 1.10–3.05) and activity level (somewhat less, OR: 1.60, 95%CI: 1.02–2.51; much less, OR: 2.49, 95%CI: 1.40–4.44), and higher odds of missed work was found (OR: 2.25, 95%CI: 1.27–3.98). There were no differences at 3 months between Asian and Black participants compared to white participants. At 6 months, Asian compared to white participants reported more fair/poor health (OR: 1.88, 95%CI: 1.13–3.12). At 6 months, Black compared to white participants reported more missed work (OR: 2.83, 95%CI: 1.60–5.00) (Figure 2, estimates reported in Supplementary Materials 5.2, 5.3).

### The relative importance of ethnicity and race in driving study outcomes compared to other covariates

The adjusted GEE models (fit using the complete dataset of SARS-CoV-2-positive and negative participants) were examined to explore associations between included covariates and study outcomes to gauge the relative importance of ethnicity and race, respectively, in driving study outcomes (Supplementary Materials 6.1–6.3 summary plots of GEE model parameter estimates). Adjusted GEE parameter estimates demonstrate a broad positive association between SARS-CoV-2-positive status, older age, female gender, any problem in social determinants of health, asthma, and lack of COVID vaccination and the 22 symptoms assessed (Supplementary Materials 6.1, 6.2 summary plots of GEE model parameter estimates). By contrast, infrequent associations between ethnicity or race and symptoms were found when adjusting for other covariates.

Examining associations of covariates that were adjusted for in the model with health status, activity level, and missed work, prominent associations were found between identifying as female or transgender, any problem in social determinants of health, asthma, and obesity and worse study outcomes (Supplementary Material 6.3 summary plot of GEE model parameter estimates).

## Discussion

In this prospective longitudinal cohort of individuals with acute SARS-CoV-2-like symptoms, there were few differences in adjusted odds of symptoms by ethnicity or race at 3 and 6 months among SARS-CoV-2-positive participants. However, there were differences in health status, activity level, and missed work. By ethnicity, at 3 months, Hispanic compared to non-Hispanic participants had worse health and lower activity levels; these differences were not present at 6 months. By race, at 3 months, Other/Multiple races compared to white participants had worse health and lower activity levels; at 6 months, these differences persisted. Additionally, at 6 months, higher odds of worse health were found for Asian participants and of missed work for Black and Other/Multiple race participants compared to white participants. The definition currently used to identify post-COVID conditions (i.e., Long COVID) is limited in scope to continuing or developing “signs, symptoms, and conditions” following acute SARS-CoV-2 infection. Notably, the differential impacts on participants’ lives by ethnicity and race identified in this study would not be captured within the current definition of post-COVID conditions. A broadening of our understanding of post-COVID conditions may be necessary to fully capture the health-related consequences of SARS-CoV-2. The strength of the association between health status, activity level, and missed work following acute SARS-CoV-2 illness and ethnicity or race is eclipsed by the strength of the association between these impacts and other determinants that we adjusted for in our model, including pre-existing health conditions and social determinants of health.

Few prior studies have evaluated longer-term sequelae of COVID-19 through the lens of ethnicity and race. Several studies used a threshold of ≥28 days to define Long COVID symptoms (28–30). We assessed the presence of SARS-CoV-2-like symptoms at least 3 months after initial infection in accordance with the current World Health Organization definition of Long COVID (38). We accounted for known ethnic and racial disparities in social determinants of health, adjusted for demographic characteristics and pre-existing health conditions, controlled for non-SARS-CoV-2 impacts through the inclusion of participants testing negative for SARS-CoV-2, and considered not only differences in persisting symptoms but other overall health measures as well. Associations between SARS-CoV-2-like symptoms and SARS-CoV-2 infection status, time point from the onset of acute symptoms, pre-existing health conditions, and lack of vaccination found in our GEE models are consistent with what is known in the literature, supporting the validity of our results.

Others have reported inconsistent associations between ethnicity and race and Long COVID symptoms. The Arizona CoVHORT study and a study of American SARS-CoV-2-positive adults who tested positive during the Omicron surge reported no significant differences by race in self-reported Long COVID symptoms (29, 30). Conversely, a U.S. Veterans Affairs EHR-based cohort study found differences between Black and white participants at 6 months, though not to the detriment of one particular group (31). The COVID States Project, a 6-weekly internet survey conducted in all 50 states and the District of Columbia between February 2021 and July 2022 (N > 16,000), found that Hispanic, Other, and white participants testing positive for SARS-CoV-2 were more likely than Asian participants to report Long COVID symptoms (32). Multiple prior studies found ethnic and racial minoritized populations to have a higher risk of Long COVID (28, 39–41). Some investigations have indicated that differences by ethnicity and race might be partially accounted for by other factors. In the RECOVER Program, a retrospective study using EHR data of participants with and without COVID-19, Black and Hispanic participants experienced higher symptom burden and a different distribution of symptoms/conditions 31–180 days after testing positive for SARS-CoV-2 than white participants (27); however, adjusting for neighborhood-level socioeconomic status attenuated several differences. In the University of California Los Angeles COVID Ambulatory Monitoring Program, a prospective cohort study of adults with SARS-CoV-2, no significant difference in symptoms 60 days after acute illness was found by ethnicity or race after adjusting for other factors, including demographic/clinical characteristics, insurance type, social vulnerability index, and baseline function (26).

Differences that we observed among ethnic and racial minoritized populations in health-related outcomes 3 and 6 months following acute SARS-CoV-2 illness might be explained by additional factors, beyond those adjusted for in this study. Several potential factors are described in the literature on health disparities. Socioeconomic deprivation has independently been associated with a higher risk of Long COVID and may mediate disparities in SARS-CoV-2 impact for ethnic and racial minoritized populations (42). Higher loss of work days may be driven by the overrepresentation of minoritized populations in physically demanding frontline industries without the option to work from home (6, 43, 44). Poor health outcomes may result from barriers to care, including inadequate health insurance and medical mistrust (6, 43–46). Mistrust and fear have been shown to deter ethnic and racial minoritized individuals with persistent SARS-CoV-2 symptoms from seeking care, compounding structural and systemic barriers (47). Ethnic and racial inequities in access to care have been illustrated in an administrative claims study, which showed that for Asian, Black, and Hispanic patients, a significantly longer time elapsed between initial infection and Long COVID diagnosis than for non-Hispanic white patients (48). Activity levels among ethnic and racial minoritized groups have been associated with differences in the built environments where they live (49–55). Finally, sequelae of COVID-19 among ethnic and racial minoritized populations may be driven by institutional, cultural, and structural racism. Experiences of discrimination adversely impact mental health and physical health through inflammation, telomere shortening, cortisol dysregulation, and increased allostatic load (56–58). Experiences of discrimination have been shown to negatively affect the quality of care and form a barrier to seeking help (59, 60). Further research is needed to understand the remaining variation in SARS-CoV-2’s impact on health status, activity level, and missed work by ethnicity and race.

Our study has several limitations. First, various ethnic and racial subgroups had small sample sizes, which reduced precision in identifying differences within these subgroups. Second, sparse data precluded adjustment for insurance and frontline worker status in GEE analysis. Third, individuals who agreed to participate in this study may not have been representative of their larger ethnic and racial subgroups. Fourth, representativeness across survey time points may have been further impacted by non-response bias. Given the variation in response rates by ethnicity and race, these limitations likely differentially impact the conclusions for specific ethnic and racial groups. Fifth, we did not evaluate important neurological and mental health sequelae of SARS-CoV-2, including cognitive impairment, difficulty concentrating, and anxiety (26, 31). Sixth, participants were recruited at different stages of the pandemic, and the ethnic and racial composition of newly recruited participants fluctuated over time (61). Heterogeneity in symptom profile and SARS-CoV-2 impact by ethnicity and race may be influenced by differences in the dominant SARS-CoV-2 variant at the time of enrollment. Finally, we did not adjust for multiple comparisons, and the generated hypotheses should be tested in confirmatory studies.

## Conclusion

Despite similar symptom prevalence, ethnic minoritized populations compared to non-Hispanic populations and racial minoritized populations compared to white populations experience more negative impacts following SARS-CoV-2 infection in terms of health status, activity level, and missed work. Increased focus on understanding drivers of ethnic and racial differences in health impacts may inform approaches to advance health equity after SARS-CoV-2 infection.

## Data availability statement

The datasets presented in this article are not readily available because the data are not approved for outside use. Requests to access the datasets should be directed to KNO, kolaugh@uw.edu.

## Ethics statement

Ethics approval of this protocol has been obtained at each individual site including Rush University (protocol number: 20030902, approved 3/14/2020), Yale University (2000027976, approved 4/30/2020), the University of Washington (UW Human Subjects Division, STUDY00009920, approved 4/2/2020), Thomas Jefferson University (20p.1150, approved 1/21/2021), the University of Texas Southwestern Medical Center (STU 2020-1352, approved 2/3/2021), the University of Texas, Houston (HSC-MS-20-0981, approved 9/10/2020), the University of California, San Francisco (20-32222, approved 1/25/2021) and the University of California, Los Angeles (20-001683, approved 12/18/2020). The Yale University ethics approval includes the role as the analytic lead. Additionally, the Rush University ethics approval includes INSPIRE data storage on the Hugo platform and transfer of data to Rush for secure storage. The studies were conducted in accordance with the local legislation and institutional requirements. The participants provided their written informed consent to participate in this study.

## Author contributions

KNO: Conceptualization, Methodology, Writing – original draft, Investigation. REK: Conceptualization, Methodology, Writing – original draft. IEM: Data curation, Formal analysis, Writing – review & editing. NLG: Conceptualization, Methodology, Writing – review & editing, Investigation. REG: Conceptualization, Methodology, Writing – review & editing, Investigation. ZZ: Conceptualization, Methodology, Writing – original draft. HY: Data curation, Formal analysis, Methodology, Writing – review & editing. S-XL: Data curation, Formal analysis, Visualization, Writing – review & editing. KCGC: Conceptualization, Methodology, Writing – review & editing. ESS: Investigation, Writing – review & editing. RCW: Investigation, Writing – review & editing. ML: Investigation, Writing – review & editing. RAW: Funding acquisition, Investigation, Writing – review & editing. IDP: Writing – review & editing. MG: Investigation, Writing – review & editing. RMH: Investigation, Writing – review & editing. MH: Writing – review & editing. JGE: Investigation, Writing – review & editing. MJH: Investigation, Writing – review & editing. MK: Investigation, Writing – review & editing. SM: Investigation, Writing – review & editing. KLR: Investigation, Writing – review & editing. RMR: Investigation, Writing – review & editing. AV: Investigation, Methodology, Writing – review & editing. AHI: Investigation, Writing – review & editing. MS: Investigation, Writing – review & editing. KK: Investigation, Writing – review & editing. SS: Conceptualization, Methodology, Writing - review & editing. GN: Conceptualization, Methodology, Writing – original draft. KAS: Conceptualization, Methodology, Writing – original draft.

## INSPIRE group

Karen Adams, BA, Regulatory Specialist, University of Washington, Clinical Core & Enrolling Site; Zohaib Ahmed, BS, Research Assistant, Rush University, Administrative Core & Enrolling Site; Grace Amadio, MD, CCRP, Research Assistant, Thomas Jefferson University, Enrolling Site; Jill Anderson, BSN, RN, Clinical Core Program Manager, University of Washington, Clinical Core & Enrolling Site; Mireya Arreguin, Research Coordinator, University of California, San Francisco, Enrolling Site; Phouthavang (Jimmie) Boliboun, BS, Research Assistant, Rush University, Administrative Core & Enrolling Site; Melissa Briggs-Hagen, MD, MPH, Centers for Disease Control and Prevention (CDC); Virginia Chan, MPH, Research Coordinator, University of California, San Francisco, Enrolling Site; Chris Chandler, BA, Research Assistant, University of California, Los Angeles, Enrolling Site; Gary Chang, PhD, Senior Biostatistician, University of Washington, Clinical Core & Enrolling Site; Alex Charlton, BS, Research Coordinator, Thomas Jefferson University, Enrolling Site; Cecilia Lara Chavez, Research Coordinator, University of California, San Francisco, Enrolling Site; David Cheng, BS, Research Coordinator, Thomas Jefferson University, Enrolling Site; Michael Choi, MBS, BA, Research Assistant, Rush University, Administrative Core & Enrolling Site; Antonia Derden, BA, Administrative Assistant, Rush University, Administrative Core & Enrolling Site; Kate Diaz Roldan, MPH, Research Assistant, University of California, Los Angeles, Enrolling Site; Jocelyn Dorney, MPH, Research Coordinator, Yale University, Analytic Core & Enrolling Site; Megan Eguchi, MPH, Data Analyst, University of California, Los Angeles, Enrolling Site; Joann G. Elmore, MD, MPH, Site Principal Investigator, University of California, Los Angeles, Enrolling Site; David Gallegos, Research Coordinator, University of Texas Southwestern Medical Center, Enrolling Site; Kristyn Gatling, MA, Research Coordinator, Rush University, Administrative Core & Enrolling Site; Caitlin A. Gaylord, Research Assistant, Rush University, Administrative Core & Enrolling Site; Nicole Gentile, MD, PhD, Co-Investigator, University of Washington, Clinical Core & Enrolling Site; Rachel E. Geyer, MPH, Research Coordinator, University of Washington, Clinical Core & Enrolling Site; Chloe Gomez, Research Assistant, Rush University, Administrative Core & Enrolling Site; Michael Gottlieb, MD, Principal Investigator, Rush University, Administrative Core & Enrolling Site; Dylan Grau, BS, Research Coordinator, Thomas Jefferson University, Enrolling Site; Diego Guzman, BS, Research Assistant, Rush University, Administrative Core & Enrolling Site; Aron J. Hall, DVM, MSPH, Centers for Disease Control and Prevention (CDC); Paavali Hannikainen, BS, Research Assistant, Thomas Jefferson University, Enrolling Site; Minna Hassaballa, BA, Research Assistant, Rush University, Administrative Core & Enrolling Site; Mandy Hill, DrPH, MPH, Site Principal Investigator, University of Texas Health Science Center at Houston, Enrolling Site; Ryan Huebinger, MD, Site Principal Investigator, University of Texas Health Science Center at Houston, Enrolling Site; Ahamed H. Idris, MD, Site Principal Investigator, University of Texas Southwestern Medical Center, Enrolling Site; Ryan Jerger, Research Assistant, Rush University, Administrative Core & Enrolling Site; Amro (Marshall) Kaadan, BS, Research Assistant, Rush University, Administrative Core & Enrolling Site; Arun Kane, BA, Research Coordinator, University of Texas Health Science Center at Houston, Enrolling Site; Efrat Kean, MD, Co-Investigator, Thomas Jefferson University, Enrolling Site; Morgan Kelly, BS, Research Coordinator, Thomas Jefferson University, Enrolling Site; Robin Kemball, MPH, Program Manager, University of California, San Francisco, Enrolling Site; Avinash Kesari, Research Assistant, Rush University, Administrative Core & Enrolling Site; Jeremiah Kinsman, MPH, NREMT, Research Manager, Yale University, Analytic Core & Enrolling Site; Robin E. Klabbers, MSc in Medicine, MSc in Global Health, Research Assistant, University of Washington, Clinical Core & Enrolling Site; Katherine Koo, MS-HSM, Program Manager, Rush University, Administrative Core & Enrolling Site; Michelle L’Hommedieu, PhD, Site Program Director, University of California, Los Angeles, Enrolling Site; Shu-Xia Li, PhD, Core Statistician, Yale University, Analytic Core & Enrolling Site; Zhenqiu Lin, PhD, Core Statistician, Yale University, Analytic Core & Enrolling Site; Mengni Liu, MS, Core Statistician, Yale University, Analytic Core & Enrolling Site; Elizabeth Lomas, Research Assistant, Rush University, Administrative Core & Enrolling Site; Victoria Lyon, MPH, Project Manager, University of Washington, Clinical Core & Enrolling Site; Zenoura Maat, Research Assistant, University of Washington, Clinical Core & Enrolling Site; Caitlin Malicki, MPH, Senior Research Manager, Yale University, Analytic Core & Enrolling Site; Kerry Malone, BA, Research Assistant, University of Washington, Clinical Core & Enrolling Site; Imtiaz Ebna Mannan, MS, Core Statistician, Yale University, Analytic Core & Enrolling Site; Riley Martin, Research Assistant, University of Texas Southwestern Medical Center, Enrolling Site; Samuel McDonald, MD, Co-Investigator, University of Texas Southwestern Medical Center, Enrolling Site; Jessica Miao, BA, Research Assistant, Thomas Jefferson University, Enrolling Site; Juan Carlos Montoy, MD, PhD, Site Principal Investigator, University of California, San Francisco, Enrolling Site; Raul Moreno, BA, Administrative Analyst, University of California, Los Angeles, Enrolling Site; Dana Morse, RN, BSN, Research Coordinator, University of Washington, Clinical Core & Enrolling Site; Graham Nichol, MD, Principal Investigator, University of Washington, Clinical Core & Enrolling Site; Peter Nikonowicz, BA, Research Coordinator, University of Texas Health Science Center at Houston, Enrolling Site; Kelli N. O’Laughlin, MD, MPH, Site Principal Investigator, University of Washington, Clinical Core & Enrolling Site; Jasmine Park, Research Assistant, University of Washington, Clinical Core & Enrolling Site; Krisna Patel, BA, Research Assistant, Rush University, Administrative Core & Enrolling Site; Ariana Pavlopoulos, Research Assistant, Rush University, Administrative Core & Enrolling Site; Senyte Pierce, Research Assistant, Yale University, Analytic Core & Enrolling Site; Ian D. Plumb, MBBS, MSc, Centers for Disease Control and Prevention (CDC); Xavier Puente, Research Assistant, Yale University, Analytic Core & Enrolling Site; Nicole Renzi, RN, Nurse Coordinator, Thomas Jefferson University, Enrolling Site; Kristin Rising, MD, MS, Site Principal Investigator, Thomas Jefferson University, Enrolling Site; Robert Rodriguez, MD, Site Principal Investigator, University of California, San Francisco, Enrolling Site; Luis Ruiz, BA, Research Assistant, University of Washington, Clinical Core & Enrolling Site; Wafa Salah, Research Assistant, Yale University, Analytic Core & Enrolling Site; Michelle Santangelo, MS, Research Manager, Rush University, Administrative Core & Enrolling Site; Sarah Sapp, MPH, Research Coordinator, University of Texas Health Science Center at Houston, Enrolling Site; Sharon Saydah, PhD, Centers for Disease Control and Prevention (CDC); Kevin Schaeffer, BS, Research Coordinator, Thomas Jefferson University, Enrolling Site; Mary Schiffgens, Grant & Finance Manager, University of Washington, Clinical Core & Enrolling Site; Hailey Shughart, BA, CCRP, Research Assistant, Thomas Jefferson University, Enrolling Site; Lindsey Shughart, BS, Research Assistant, Thomas Jefferson University, Enrolling Site; Carly Shutty, BSN, Research Coordinator, Thomas Jefferson University, Enrolling Site; Erica S. Spatz, MD, MHS, Principal Investigator, Yale University, Analytic Core & Enrolling Site; Kari A. Stephens, PhD, MS, Principal Investigator, University of Washington, Clinical Core & Enrolling Site; Tracy Stober, BA, MA, Patient Representative, University of Washington, Clinical Core & Enrolling Site; Andrew Ulrich, MD, Co-Investigator, Yale University, Analytic Core & Enrolling Site; Arjun Venkatesh, MD, MBA, MHS, Principal Investigator, Yale University, Analytic Core & Enrolling Site; Ralph C. Wang, MD, MAS, Site Principal Investigator, University of California, San Francisco, Enrolling Site; Phillip Watts, BA, MM, CCRP, Research Coordinator, Thomas Jefferson University, Enrolling Site; Robert A. Weinstein, MD, Principal Investigator, Rush University, Administrative Core & Enrolling Site; Michael Willis, AS, BSHS, Research Coordinator, University of Washington, Clinical Core & Enrolling Site; Lauren E. Wisk, PhD, Co-Investigator, University of California, Los Angeles, Enrolling Site; Angela Wong, BA, Research Coordinator, University of California, San Francisco, Enrolling Site; Geoffrey Yang, BA, Research Assistant, Rush University, Administrative Core & Enrolling Site; Zimo Yang, MS, Core Statistician, Yale University, Analytic Core & Enrolling Site; Huihui Yu, PhD, Core Statistician, Yale University, Analytic Core & Enrolling Site; Zihan Zhang, Analyst, University of Washington, Clinical Core & Enrolling Site.
