# Supplementary material for: Ethnic and racial differences in self-reported symptoms, health status, activity level, and missed work at 3 and 6 months following SARS-CoV-2 infection
Source: Front Public Health. 2024 Jan 30;11:1324636. doi: 10.3389/fpubh.2023.1324636 (PMC10861779; doi:10.3389/fpubh.2023.1324636)

**List of Appendices**

- Supplementary Material 1. Categories entered into the generalized estimating equation (GEE) models
- Supplementary Material 2. Demonstration of OR estimation from GEE modeling
- Supplementary Material 3. Follow-up survey response status by ethnicity and race
- Supplementary Material 4. Symptoms, health, activity level, and missed work over time among adult INSPIRE SARS-CoV-2-egative participants by ethnicity and race
- Supplementary Material 5.1a. GEE adjusted OR output for ethnicity among SARS-CoV-2-positive INSPIRE participants (ref: Non-Hispanic)
- Supplementary Material 5.1b. GEE adjusted OR output for ethnicity among SARS-CoV-2-negative INSPIRE participants (ref: Non-Hispanic)
- Supplementary Material 5.2a. GEE adjusted OR output for race at 3-month among SARS-CoV-2-positive INSPIRE participants (ref: White)
- Supplementary Material 5.2b. GEE adjusted OR output for race at 3-month among SARS-CoV-2-negative INSPIRE participants (ref: White)
- Supplementary Material 5.3a. GEE adjusted OR output for race at 6-month among SARS-CoV-2-positive INSPIRE participants (ref: White)
- Supplementary Material Table 5.3b. GEE adjusted OR output for race at 6-month among SARS-CoV-2-negative INSPIRE participants (ref: White)
- Supplementary Material 6.1. INSPIRE summary plot of model parameter estimates for the association of ethnicity and symptoms
- Supplementary Material 6.2. INSPIRE summary plot of model parameter estimates for the association of race and symptoms
- Supplementary Material 6.3. INSPIRE summary plot of model parameter estimates for the association of ethnicity/race and health status, activity level, and missed work.

**Supplementary Material 1. Categories entered into the generalized estimating equation (GEE) models**

| **Covariate** | **Survey question** | **Collapsed Categories** |
| --- | --- | --- |
| **Any social determinants of health problem (any problem/no problem)** | | |
| Housing insecurity | What is your living situation today? | **Any problem**   - I have a place to live today, but I am worried about losing it in the future. - I do not have a steady place to live (I am temporarily staying with others, in a hotel, in a shelter, living outside on the street, on a beach, in a car, abandoned building, bus or train station, or in a park.   **No problem**   - I have a steady place to live |
| Food insecurity | In the past month, how often was the following true: "you worried that your food would run out before you got money to buy more." | **Any problem**   - Sometimes true - Often true   **No problem**   - Never true |
| Utility access | In the past month, has the electric, gas, oil, or water company shut off services (e.g., sending a warning notice) in your home? | **Any problem**   - Threatened to shut off services - Already shut off services   **No problem**   - Never shut off services |
| Transportation access | In the past month, has lack of reliable transportation kept you from medical appointments, meetings, work, or from getting to things needed for daily living? (Check all that apply) | **Any problem**   - It has kept me from medical appointments or getting medications - It has kept me from non-medical meetings, non-medical appointments, work, or getting things that I need   **No problem**   - It has not kept me from getting things I need |
| **Moderate to extreme substance use past 12 months (yes/no)** | | |
| Excessive alcohol use | In the past 12 months, how often have you had 5 or more drinks (men)/4 or more drinks (women) containing alcohol in one day? | **Moderate to extreme problem**   - Daily or nearly daily - Weekly   **No to mild problem**   - Less than monthly - Monthly - Not at all |
| Prescription abuse | In the past 12 months, how often have you used any prescription medications just for the feeling, more than prescribed or that were not prescribed for you? | **Moderate to extreme problem**   - Daily or nearly daily - Weekly - Less than monthly - Monthly   **No to mild problem**   - Not at all |
| Marijuana use | In the past 12 months, how often have you smoked or vaped marijuana? | **Moderate to extreme problem**   - Daily or nearly daily - Weekly   **No to mild problem**   - Less than monthly - Monthly - Not at all |
| Drug use | In the past 12 months, how often have you used any drugs including cocaine or crack, heroin, methamphetamine (crystal meth), hallucinogens, ecstasy/MDMA? | **Moderate to extreme problem**   - Daily or nearly daily - Weekly - Less than monthly - Monthly   **No to mild problem**   - Not at all |
|  | | |
| Education | What is the highest level of education you have completed? | **High school or less**   - Less than high school - High school graduate   **Some college or 2-year degree**   - Some college - 2-year degree   **4 or more years of college**   - 4-year degree - More than 4 years |
| Moderate to extreme tobacco or nicotine use past 12 months (yes/no) | In the past 12 months, how often have you used tobacco or any other nicotine product (i.e., e-cigarette, vaping, or chewing tobacco)? | **Moderate to extreme problem**   - Daily or nearly daily - Weekly - Less than monthly - Monthly   **No to mild problem**   - Not at all |

**Supplementary Material 2. Demonstration of OR estimation from GEE modeling**

Example of calculating the marginal race effects on each of the 29 binary outcomes at a time point:

In the GEE models using the LOGIT link, the logit function of probability of having an outcome (e.g., wheezing), for patient $i$ at survey time $t$ *,* ${\mu_{it},}$is estimated as below.

**
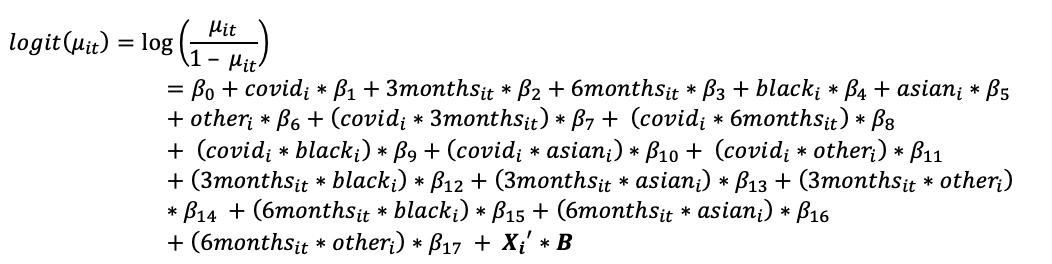
**

Where we have included interactions between 1) race groups and COVID-19 status, 2) race groups and survey time points, and 3) COVID-19 status and survey time pints; $\boldsymbol{X}_{\boldsymbol{i}}$ denotes a vector of observed values of 24 time-invariant risk variables for patient $i$, $\boldsymbol{B}$ denotes these covariates’ coefficients for outcome (like wheezing), and

${covid}_{it}=\left\{ \begin{aligned} 1, &if COVID+ \\ 0, &else \end{aligned} \right.$ ;

${3months}_{i}=\left\{ \begin{aligned} 1, &if survey taken at 3 months \\ 0, &else \end{aligned} \right.$ ;

${6months}_{i}=\left\{ \begin{aligned} 1, &if survey taken at 6 months \\ 0, &else \end{aligned} \right.$ ;

$${asian}_{it}=\left\{ \begin{aligned} 1, &if race is Asian \\ 0, &else \end{aligned} \right.$$

${black}_{it}=\left\{ \begin{aligned} 1, &if race is Black \\ 0, &else \end{aligned} \right.$ ;

$${other}_{it}=\left\{ \begin{aligned} 1, &if race is Other/Multiple \\ 0, &else \end{aligned} \right.$$

The estimated coefficients from the GEE modeling are used to calculate the marginal odds ratio. For example, the marginal odds ratio of black covid-positive participants experiencing wheezing at 3 months in comparison to White covid-positive participants at 3 months is calculated as below:

**
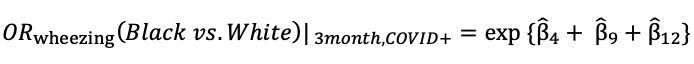
**

**Supplementary Material 3.**  **Follow-up survey response status by ethnicity and race**

|  | 3-month responders/3-month survey recipients | p-value | 6-month responders/6-month survey recipients | p-value |
| --- | --- | --- | --- | --- |
| **Ethnicity** |  |  |  |  |
| Hispanic | 450/656 (68.6%) | **<0.001** | 231/287 (80.5%) | 0.052 |
| Non-Hispanic | 2644/3508 (75.4%) |  | 1510/1777 (85.0%) |  |
| **Race** |  |  |  |  |
| Asian | 368/452 (81.4%) | **<0.001** | 191/209 (91.4%) | **<0.001** |
| Black | 283/505 (56.0%) |  | 145/208 (69.7%) |  |
| Other/Multiple | 291/395 (73.7%) |  | 158/192 (82.3%) |  |
| White | 2129/2762 (77.1%) |  | 1239/1444 (85.8%) |  |

Note: All participants who completed the enrollment survey and did not develop new COVID-19 infection by the 3-month follow-up time point received the 3-month survey. All participants who completed the enrollment and 3-month survey and did not develop new COVID-19 infection by the 6-month follow-up time point received the 6-month survey.

**Supplementary Material 4. Symptoms, health, activity level, and missed work over time among adult INSPIRE SARS-CoV-2-negative participants by ethnicity and race**

|  | **Enrollment^a^** | | | | | | **3-Month^b^** | | | | | | **6-Month^c^** | | | | | |
| --- | --- | --- | --- | --- | --- | --- | --- | --- | --- | --- | --- | --- | --- | --- | --- | --- | --- | --- |
|  | **Ethnicity** | | **Race** | | | | **Ethnicity** | | **Race** | | | | **Ethnicity** | | **Race** | | | |
|  | ***Hispanic***  ***(N=132)*** | ***Non-Hispanic***  ***(N=669)*** | **Asian (N=117)** | **Black (N=104)** | **Other/ Multiple (N=64)** | **White (N=507)** | **Hispanic  (N=120)** | **Non-Hispanic  (N=620)** | **Asian (N=110)** | **Black (N=97)** | **Other/ Multiple (N=59)** | **White (N=464)** | **Hispanic  (N=57)** | **Non-Hispanic (N=302)** | **Asian (N=49)** | **Black (N=36)** | **Other/ Multiple (N=34)** | **White (N=239)** |
| Symptom Category | N (%) | N (%) | N (%) | N (%) | N (%) | N (%) | N (%) | N (%) | N (%) | N (%) | N (%) | N (%) | N (%) | N (%) | N (%) | N (%) | N (%) | N (%) |
| Constitutional |  |  |  |  |  |  |  |  |  |  |  |  |  |  |  |  |  |  |
| Tired | 65 (50.4) | 366 (55.4) | 59 (50.9) | 42 (40.8) | 39 (62.9) | 286 (57.2) | 19 (15.8) | 110 (17.8) | 13 (11.8) | 12 (12.4) | 13 (22.4) | 89 (19.2) | 11 (19.6) | 50 (16.7) | 8 (16.3) | 4 (11.1) | 4 (12.1) | 43 (18.1) |
| Chills | 37 (28.7) | 178 (26.9) | 25 (21.6) | 25 (24.3) | 19 (30.6) | 145 (29.0) | 7 (5.8) | 34 (5.5) | 3 (2.7) | 5 (5.2) | 5 (8.6) | 27 (5.8) | 8 (14.3) | 15 (5.0) | 3 (6.1) | 3 (8.3) | 1 (3.0) | 14 (5.9) |
| Feeling Hot | 26 (20.2) | 166 (25.1) | 19 (16.4) | 26 (25.2) | 14 (22.6) | 134 (26.8) | 5 (4.2) | 26 (4.2) | 3 (2.7) | 5 (5.2) | 3 (5.2) | 19 (4.1) | 3 (5.4) | 11 (3.7) | 0 (0.0) | 2 (5.6) | 2 (6.1) | 9 (3.8) |
| Fever | 14 (10.9) | 91 (13.8) | 10 (8.6) | 13 (12.6) | 9 (14.5) | 74 (14.8) | 0 (0.0) | 20 (3.2) | 1 (0.9) | 1 (1.0) | 1 (1.7) | 17 (3.7) | 5 (8.9) | 4 (1.3) | 0 (0.0) | 1 (2.8) | 1 (3.0) | 6 (2.5) |
| Shakes | 11 (8.5) | 40 (6.1) | 5 (4.3) | 14 (13.6) | 5 (8.1) | 27 (5.4) | 1 (0.8) | 12 (1.9) | 0 (0.0) | 3 (3.1) | 1 (1.7) | 9 (1.9) | 2 (3.6) | 3 (1.0) | 0 (0.0) | 1 (2.8) | 0 (0.0) | 3 (1.3) |
| HEENT^d^ |  |  |  |  |  |  |  |  |  |  |  |  |  |  |  |  |  |  |
| Headache | 61 (47.3) | 298 (45.1) | 47 (40.5) | 39 (37.9) | 33 (53.2) | 238 (47.6) | 18 (15.0) | 81 (13.1) | 11 (10.0) | 10 (10.3) | 9 (15.5) | 69 (14.9) | 14 (25.0) | 33 (11.0) | 5 (10.2) | 3 (8.3) | 3 (9.1) | 33 (13.9) |
| Runny Nose | 67 (51.9) | 304 (46.0) | 44 (37.9) | 36 (35.0) | 30 (48.4) | 256 (51.2) | 14 (11.7) | 62 (10.0) | 10 (9.1) | 5 (5.2) | 8 (13.8) | 52 (11.2) | 10 (17.9) | 32 (10.7) | 4 (8.2) | 5 (13.9) | 3 (9.1) | 28 (11.8) |
| Loss of smell | 10 (7.8) | 44 (6.7) | 4 (3.4) | 12 (11.7) | 2 (3.2) | 38 (7.6) | 3 (2.5) | 13 (2.1) | 1 (0.9) | 0 (0.0) | 0 (0.0) | 15 (3.2) | 3 (5.4) | 6 (2.0) | 2 (4.1) | 1 (2.8) | 1 (3.0) | 6 (2.5) |
| Loss of taste | 10 (7.8) | 51 (7.7) | 4 (3.4) | 17 (16.5) | 3 (4.8) | 39 (7.8) | 3 (2.5) | 19 (3.1) | 1 (0.9) | 5 (5.2) | 0 (0.0) | 15 (3.2) | 3 (5.4) | 8 (2.7) | 2 (4.1) | 3 (8.3) | 1 (3.0) | 5 (2.1) |
| Sore throat | 70 (54.3) | 299 (45.2) | 52 (44.8) | 35 (34.0) | 31 (50.0) | 246 (49.2) | 12 (10.0) | 51 (8.2) | 9 (8.2) | 8 (8.2) | 6 (10.3) | 40 (8.6) | 12 (21.4) | 24 (8.0) | 3 (6.1) | 2 (5.6) | 3 (9.1) | 25 (10.5) |
| Loss of hair | 4 (3.1) | 14 (2.1) | 3 (2.6) | 3 (2.9) | 2 (3.2) | 9 (1.8) | 5 (4.2) | 26 (4.2) | 5 (4.5) | 6 (6.2) | 0 (0.0) | 17 (3.7) | 1 (1.8) | 12 (4.0) | 2 (4.1) | 1 (2.8) | 0 (0.0) | 9 (3.8) |
| Pulmonary |  |  |  |  |  |  |  |  |  |  |  |  |  |  |  |  |  |  |
| Cough | 40 (31.0) | 208 (31.5) | 29 (25.0) | 31 (30.1) | 20 (32.3) | 164 (32.8) | 7 (5.8) | 38 (6.1) | 7 (6.4) | 6 (6.2) | 2 (3.4) | 30 (6.5) | 8 (14.3) | 18 (6.0) | 2 (4.1) | 3 (8.3) | 2 (6.1) | 18 (7.6) |
| Shortness of breath | 19 (14.7) | 116 (17.5) | 12 (10.3) | 21 (20.4) | 12 (19.4) | 89 (17.8) | 7 (5.8) | 31 (5.0) | 4 (3.6) | 4 (4.1) | 3 (5.2) | 26 (5.6) | 6 (10.7) | 15 (5.0) | 1 (2.0) | 4 (11.1) | 1 (3.0) | 15 (6.3) |
| Wheezing | 10 (7.8) | 47 (7.1) | 4 (3.4) | 9 (8.7) | 4 (6.5) | 39 (7.8) | 5 (4.2) | 13 (2.1) | 1 (0.9) | 3 (3.1) | 2 (3.4) | 11 (2.4) | 2 (3.6) | 9 (3.0) | 0 (0.0) | 3 (8.3) | 1 (3.0) | 6 (2.5) |
| Cardiovascular |  |  |  |  |  |  |  |  |  |  |  |  |  |  |  |  |  |  |
| Chest pains | 17 (13.2) | 86 (13.0) | 11 (9.5) | 16 (15.5) | 7 (11.3) | 67 (13.4) | 1 (0.8) | 23 (3.7) | 3 (2.7) | 2 (2.1) | 2 (3.4) | 17 (3.7) | 6 (10.7) | 12 (4.0) | 1 (2.0) | 3 (8.3) | 1 (3.0) | 12 (5.1) |
| Palpitations | 12 (9.3) | 41 (6.2) | 5 (4.3) | 7 (6.8) | 6 (9.7) | 36 (7.2) | 4 (3.3) | 24 (3.9) | 5 (4.5) | 3 (3.1) | 1 (1.7) | 18 (3.9) | 4 (7.1) | 10 (3.3) | 1 (2.0) | 2 (5.6) | 1 (3.0) | 9 (3.8) |
| Gastrointestinal |  |  |  |  |  |  |  |  |  |  |  |  |  |  |  |  |  |  |
| Diarrhea | 22 (17.1) | 100 (15.1) | 12 (10.3) | 22 (21.4) | 11 (17.7) | 76 (15.2) | 6 (5.0) | 30 (4.8) | 1 (0.9) | 5 (5.2) | 4 (6.9) | 25 (5.4) | 4 (7.1) | 8 (2.7) | 1 (2.0) | 2 (5.6) | 1 (3.0) | 8 (3.4) |
| Nausea or vomiting | 10 (7.8) | 92 (13.9) | 4 (3.4) | 22 (21.4) | 8 (12.9) | 66 (13.2) | 4 (3.3) | 24 (3.9) | 1 (0.9) | 6 (6.2) | 2 (3.4) | 19 (4.1) | 3 (5.4) | 10 (3.3) | 4 (8.2) | 2 (5.6) | 0 (0.0) | 7 (3.0) |
| Abdominal pain | 14 (10.9) | 53 (8.0) | 5 (4.3) | 13 (12.6) | 5 (8.1) | 42 (8.4) | 6 (5.0) | 19 (3.1) | 2 (1.8) | 4 (4.1) | 4 (6.9) | 15 (3.2) | 1 (1.8) | 10 (3.3) | 2 (4.1) | 2 (5.6) | 0 (0.0) | 7 (3.0) |
| Musculoskeletal |  |  |  |  |  |  |  |  |  |  |  |  |  |  |  |  |  |  |
| Aches | 52 (40.3) | 221 (33.4) | 35 (30.2) | 35 (34.0) | 25 (40.3) | 176 (35.2) | 8 (6.7) | 74 (12.0) | 13 (11.8) | 7 (7.2) | 4 (6.9) | 57 (12.3) | 8 (14.3) | 34 (11.3) | 7 (14.3) | 4 (11.1) | 4 (12.1) | 25 (10.5) |
| Joint pains | 27 (20.9) | 123 (18.6) | 9 (7.8) | 20 (19.4) | 18 (29.0) | 100 (20.0) | 8 (6.7) | 62 (10.0) | 7 (6.4) | 7 (7.2) | 4 (6.9) | 50 (10.8) | 7 (12.5) | 24 (8.0) | 2 (4.1) | 3 (8.3) | 2 (6.1) | 23 (9.7) |
| Symptom summary |  |  |  |  |  |  |  |  |  |  |  |  |  |  |  |  |  |  |
| Other symptoms | 10 (7.8) | 40 (6.1) | 7 (6.0) | 3 (2.9) | 3 (4.8) | 37 (7.4) | 3 (2.5) | 20 (3.2) | 2 (1.8) | 2 (2.1) | 1 (1.7) | 19 (4.1) | 3 (5.4) | 6 (2.0) | 2 (4.1) | 0 (0.0) | 1 (3.0) | 7 (3.0) |
| ≥3 symptoms (not including other) | 84 (65.1) | 410 (62.0) | 62 (53.4) | 59 (57.3) | 40 (64.5) | 325 (65.0) | 23 (19.2) | 107 (17.3) | 17 (15.5) | 12 (12.4) | 13 (22.4) | 87 (18.8) | 15 (26.8) | 54 (18.0) | 7 (14.3) | 6 (16.7) | 5 (15.2) | 48 (20.3) |
| No symptoms | 16 (12.1) | 104 (15.5) | 15 (12.8) | 24 (23.1) | 8 (12.5) | 70 (13.8) | 82 (68.3) | 422 (68.2) | 82 (74.5) | 66 (68.0) | 36 (62.1) | 314 (67.7) | 33 (58.9) | 216 (72.0) | 37 (75.5) | 27 (75.0) | 23 (69.7) | 163 (68.8) |
| Health status |  |  |  |  |  |  |  |  |  |  |  |  |  |  |  |  |  |  |
| Excellent |  |  |  |  |  |  | 8 (6.8) | 32 (5.2) | 4 (3.7) | 7 (7.2) | 2 (3.5) | 25 (5.4) | 5 (8.9) | 17 (5.8) | 5 (10.9) | 4 (11.8) | 2 (5.9) | 10 (4.3) |
| Very good |  |  |  |  |  |  | 22 (18.6) | 139 (22.6) | 25 (22.9) | 19 (19.6) | 8 (14.0) | 111 (24.1) | 9 (16.1) | 70 (23.8) | 6 (13.0) | 10 (29.4) | 8 (23.5) | 57 (24.3) |
| Good |  |  |  |  |  |  | 41 (34.7) | 230 (37.3) | 49 (45.0) | 32 (33.0) | 20 (35.1) | 166 (36.0) | 27 (48.2) | 107 (36.4) | 19 (41.3) | 11 (32.4) | 12 (35.3) | 92 (39.1) |
| Fair |  |  |  |  |  |  | 37 (31.4) | 159 (25.8) | 26 (23.9) | 28 (28.9) | 22 (38.6) | 118 (25.6) | 8 (14.3) | 78 (26.5) | 13 (28.3) | 5 (14.7) | 8 (23.5) | 60 (25.5) |
| Poor |  |  |  |  |  |  | 6 (5.1) | 53 (8.6) | 5 (4.6) | 11 (11.3) | 4 (7.0) | 38 (8.2) | 4 (7.1) | 20 (6.8) | 3 (6.5) | 3 (8.8) | 4 (11.8) | 14 (6.0) |
| Do not know |  |  |  |  |  |  | 4 (3.4) | 2 (0.3) | 0 (0.0) | 0 (0.0) | 1 (1.8) | 3 (0.7) | 1 (1.8) | 1 (0.3) | 0 (0.0) | 0 (0.0) | 0 (0.0) | 2 (0.9) |
| No answer |  |  |  |  |  |  | 0 (0.0) | 1 (0.2) | 0 (0.0) | 0 (0.0) | 0 (0.0) | 0 (0.0) | 2 (3.6) | 1 (0.3) | 0 (0.0) | 1 (2.9) | 0 (0.0) | 0 (0.0) |
| Activity level^e^ |  |  |  |  |  |  |  |  |  |  |  |  |  |  |  |  |  |  |
| Same as before |  |  |  |  |  |  | 77 (65.3) | 431 (70.7) | 80 (73.4) | 71 (73.2) | 39 (68.4) | 313 (68.9) | 37 (66.1) | 194 (66.4) | 29 (63.0) | 25 (73.5) | 23 (67.6) | 155 (66.5) |
| Somewhat less than before |  |  |  |  |  |  | 24 (20.3) | 120 (19.7) | 23 (21.1) | 13 (13.4) | 13 (22.8) | 92 (20.3) | 13 (23.2) | 67 (22.9) | 13 (28.3) | 6 (17.6) | 7 (20.6) | 53 (22.7) |
| Much less than before |  |  |  |  |  |  | 17 (14.4) | 59 (9.7) | 6 (5.5) | 13 (13.4) | 5 (8.8) | 49 (10.8) | 6 (10.7) | 31 (10.6) | 4 (8.7) | 3 (8.8) | 4 (11.8) | 25 (10.7) |
| Missed work past 3 months due to health reasons |  |  |  |  |  |  |  |  |  |  |  |  |  |  |  |  |  |  |
| 0-5 workdays |  |  |  |  |  |  | 73 (61.9) | 381 (61.9) | 72 (66.1) | 47 (48.5) | 36 (63.2) | 296 (64.2) | 37 (66.1) | 178 (60.5) | 30 (65.2) | 14 (41.2) | 22 (64.7) | 149 (63.4) |
| 6-10 workdays |  |  |  |  |  |  | 10 (8.5) | 27 (4.4) | 2 (1.8) | 7 (7.2) | 5 (8.8) | 22 (4.8) | 6 (10.7) | 9 (3.1) | 0 (0.0) | 3 (8.8) | 4 (11.8) | 8 (3.4) |
| 11-20 workdays |  |  |  |  |  |  | 3 (2.5) | 15 (2.4) | 1 (0.9) | 6 (6.2) | 0 (0.0) | 11 (2.4) | 2 (3.6) | 3 (1.0) | 0 (0.0) | 1 (2.9) | 0 (0.0) | 4 (1.7) |
| up to 4 weeks |  |  |  |  |  |  | 4 (3.4) | 15 (2.4) | 3 (2.8) | 3 (3.1) | 2 (3.5) | 10 (2.2) | 1 (1.8) | 13 (4.4) | 3 (6.5) | 2 (5.9) | 1 (2.9) | 9 (3.8) |
| Don't work |  |  |  |  |  |  | 28 (23.7) | 178 (28.9) | 31 (28.4) | 34 (35.1) | 14 (24.6) | 122 (26.5) | 10 (17.9) | 91 (31.0) | 13 (28.3) | 14 (41.2) | 7 (20.6) | 65 (27.7) |

Footnotes:

^a^Included COVID-negative participants who completed both baseline and 3 months surveys; excluded participants with missing ethnicity (n=20)/missing race (n=29) and with missing symptom responses (n=11).

^b^Included COVID-negative participants who completed both baseline and 3 months surveys; excluded participants with negative index COVID-19 diagnosis and subsequent positive COVID-19 diagnosis at 3 months (n=62); further excluded participants with missing ethnicity (n=19)/missing race (n=29) and with missing symptom responses (n=1).

^c^Included included COVID-positive participants who completed baseline, 3 months, and 6 months surveys; excluded participants with negative index COVID-19 diagnosis and subsequent positive COVID-19 diagnosis at 3/6 months (n=62); further excluded participants with missing ethnicity (n=7)/missing race (n=8) and with missing symptom responses (n=3).

^d^HEENT = head, ears, eyes, nose, throat

^e^Excluded participants with missing active level variables (n=13 for 3 months; n=11 for 6 months), or missed work (n=6 for 3 months; n=9 for 6 months) , or health (n=6 for 3 months; n=9 for 6 months)

**Supplementary Material 5.1a. GEE adjusted OR output for ethnicity among SARS-CoV-2-positive INSPIRE participants (ref: Non-Hispanic)**

|  | 3-month | | 6-month | |
| --- | --- | --- | --- | --- |
| Outcome | Estimated **adjusted** OR (95% CI) | p-value | Estimated **adjusted** OR (95% CI) | p-value |
| Constitutional |  |  |  |  |
| Tired | 1.00 (0.71, 1.39) | 0.98 | 1.27 (0.80, 2.02) | 0.31 |
| Chills | 1.57 (0.99, 2.49) | 0.05 | 1.42 (0.72, 2.81) | 0.31 |
| Feeling hot | 1.15 (0.68, 1.95) | 0.59 | 1.16 (0.56, 2.39) | 0.69 |
| Fever | 1.13 (0.56, 2.26) | 0.73 | 1.83 (0.72, 4.62) | 0.20 |
| Shakes | 1.21 (0.53, 2.76) | 0.64 | 1.61 (0.49, 5.25) | 0.43 |
| HEENT^+^ |  |  |  |  |
| Headache | 1.70 (1.20, 2.42) | <.01^**^ | 1.97 (1.25, 3.11) | <.01^**^ |
| Runny nose | 0.99 (0.64, 1.55) | 0.97 | 0.78 (0.43, 1.44) | 0.43 |
| Loss of smell | 0.92 (0.61, 1.38) | 0.69 | 0.70 (0.35, 1.41) | 0.32 |
| Loss of taste | 0.89 (0.57, 1.39) | 0.62 | 0.62 (0.28, 1.39) | 0.24 |
| Sore throat | 1.08 (0.68, 1.73) | 0.74 | 1.47 (0.84, 2.57) | 0.18 |
| Loss of hair | 1.61 (0.94, 2.75) | 0.08 | 1.44 (0.75, 2.76) | 0.27 |
| Pulmonary |  |  |  |  |
| Cough | 1.06 (0.65, 1.74) | 0.80 | 1.11 (0.58, 2.13) | 0.76 |
| Shortness of breath | 1.29 (0.83, 2.02) | 0.26 | 1.29 (0.73, 2.31) | 0.38 |
| Wheezing | 1.30 (0.60, 2.85) | 0.50 | 1.11 (0.36, 3.40) | 0.86 |
| Cardiovascular |  |  |  |  |
| Chest pains | 0.95 (0.51, 1.76) | 0.86 | 1.20 (0.56, 2.57) | 0.64 |
| Palpitations | 1.75 (0.93, 3.28) | 0.08 | 1.63 (0.73, 3.65) | 0.23 |
| Gastrointestinal |  |  |  |  |
| Diarrhea | 1.40 (0.76, 2.58) | 0.27 | 1.74 (0.77, 3.95) | 0.18 |
| Nausea or vomiting | 1.65 (0.86, 3.18) | 0.13 | 2.20 (1.02, 4.77) | 0.05^*^ |
| Abdominal pain | 1.22 (0.60, 2.47) | 0.59 | 1.54 (0.67, 3.56) | 0.31 |
| Musculoskeletal |  |  |  |  |
| Aches | 1.22 (0.82, 1.80) | 0.33 | 1.19 (0.73, 1.95) | 0.48 |
| Joint pains | 1.22 (0.81, 1.84) | 0.35 | 1.05 (0.60, 1.82) | 0.87 |
| Symptom summary |  |  |  |  |
| Other symptoms | 1.28 (0.68, 2.39) | 0.44 | 2.32 (1.14, 4.72) | 0.02^*^ |
| ≥3 symptoms (not including other) | 1.23 (0.89, 1.71) | 0.21 | 1.35 (0.89, 2.05) | 0.15 |
| No symptoms | 0.77 (0.58, 1.03) | 0.08 | 0.81 (0.57, 1.15) | 0.23 |
| Activity level (ref: same as before) |  |  |  |  |
| Somewhat less than before | 1.47 (1.06, 2.02) | 0.02^*^ | 1.40 (0.94, 2.10) | 0.10 |
| Much less than before | 2.23 (1.38, 3.61) | <.01^**^ | 1.75 (0.98, 3.12) | 0.06 |
| Missed work due to health reasons past 3 months (ref: 0-5 workdays) |  |  |  |  |
| Missed >5 workdays | 1.11 (0.75, 1.63) | 0.60 | 1.34 (0.79, 2.29) | 0.28 |
| Health status (ref: good) |  |  |  |  |
| Excellent/Very good | 1.20 (0.81, 1.76) | 0.36 | 1.24 (0.81, 1.92) | 0.33 |
| Fair/Poor | 1.94 (1.36, 2.78) | <.01^**^ | 1.25 (0.78, 1.98) | 0.35 |

^*^p-value <.05; ^**^p-value < .01; ^+^Head, ears, eyes, nose, throat

**Supplementary Material 5.1b. GEE adjusted OR output for ethnicity among SARS-CoV-2-negative INSPIRE participants (ref: Non-Hispanic)**

|  | 3-month | | 6-month | |
| --- | --- | --- | --- | --- |
| Outcome | Estimated **adjusted** OR (95% CI) | p-value | Estimated **adjusted** OR (95% CI) | p-value |
| Constitutional |  |  |  |  |
| Tired | 0.73 (0.44, 1.19) | 0.2 | 0.89 (0.51, 1.55) | 0.68 |
| Chills | 1.29 (0.74, 2.23) | 0.37 | 1.16 (0.55, 2.46) | 0.69 |
| Feeling hot | 0.72 (0.34, 1.54) | 0.4 | 0.72 (0.29, 1.81) | 0.49 |
| Fever | 0.91 (0.42, 1.94) | 0.8 | 1.47 (0.47, 4.55) | 0.51 |
| Shakes | 0.88 (0.34, 2.24) | 0.78 | 1.16 (0.30, 4.41) | 0.83 |
| HEENT^+^ |  |  |  |  |
| Headache | 1.29 (0.82, 2.02) | 0.27 | 1.49 (0.88, 2.52) | 0.14 |
| Runny nose | 1.50 (0.89, 2.52) | 0.13 | 1.18 (0.60, 2.33) | 0.63 |
| Loss of smell | 0.97 (0.39, 2.39) | 0.94 | 0.74 (0.24, 2.29) | 0.6 |
| Loss of taste | 0.52 (0.21, 1.29) | 0.16 | 0.36 (0.11, 1.22) | 0.1 |
| Sore throat | 1.41 (0.82, 2.43) | 0.21 | 1.91 (0.98, 3.75) | 0.06 |
| Loss of hair | 0.24 (0.05, 1.07) | 0.06 | 0.21 (0.05, 0.98) | 0.05 |
| Pulmonary |  |  |  |  |
| Cough | 1.34 (0.72, 2.49) | 0.35 | 1.39 (0.64, 3.04) | 0.4 |
| Shortness of breath | 0.77 (0.37, 1.57) | 0.47 | 0.77 (0.33, 1.80) | 0.54 |
| Wheezing | 1.22 (0.41, 3.61) | 0.73 | 1.03 (0.29, 3.63) | 0.96 |
| Cardiovascular |  |  |  |  |
| Chest pains | 0.90 (0.44, 1.85) | 0.77 | 1.14 (0.45, 2.87) | 0.79 |
| Palpitations | 0.70 (0.29, 1.69) | 0.43 | 0.66 (0.23, 1.89) | 0.43 |
| Gastrointestinal |  |  |  |  |
| Diarrhea | 1.25 (0.60, 2.61) | 0.55 | 1.55 (0.62, 3.92) | 0.35 |
| Nausea or vomiting | 0.41 (0.15, 1.15) | 0.09 | 0.55 (0.18, 1.68) | 0.3 |
| Abdominal pain | 1.28 (0.49, 3.38) | 0.61 | 1.63 (0.62, 4.28) | 0.32 |
| Musculoskeletal |  |  |  |  |
| Aches | 1.06 (0.65, 1.72) | 0.81 | 1.04 (0.57, 1.89) | 0.9 |
| Joint pains | 1.04 (0.58, 1.87) | 0.88 | 0.90 (0.45, 1.79) | 0.76 |
| Symptom summary |  |  |  |  |
| Other symptoms | 1.10 (0.47, 2.58) | 0.83 | 2.00 (0.77, 5.17) | 0.15 |
| ≥3 symptoms (not including other) | 1.08 (0.68, 1.73) | 0.73 | 1.19 (0.69, 2.06) | 0.53 |
| No symptoms | 1.10 (0.69, 1.73) | 0.69 | 1.15 (0.69, 1.91) | 0.60 |
| Activity Level (ref: same as before) |  |  |  |  |
| Somewhat less than before | 0.80 (0.41, 1.56) | 0.50 | 0.83 (0.44, 1.57) | 0.57 |
| Much less than before | 0.94 (0.41, 2.13) | 0.88 | 1.20 (0.56, 2.57) | 0.64 |
| Missed work due to health reasons past 3 months (ref: 0-5 workdays) |  |  |  |  |
| Missed >5 workdays | 1.15 (0.53, 2.50) | 0.73 | 0.95 (0.48, 1.88) | 0.88 |
| Health status (ref: good) |  |  |  |  |
| Excellent/Very good | 0.92 (0.47, 1.82) | 0.81 | 0.89 (0.45, 1.73) | 0.72 |
| Fair/Poor | 0.67 (0.36, 1.23) | 0.20 | 1.04 (0.60, 1.79) | 0.89 |

^*^p-value <.05; ^**^p-value < .01; ^+^Head, ears, eyes, nose, throat

**Supplementary Material 5.2a. GEE adjusted OR output for race at 3-month among SARS-CoV-2-positive INSPIRE participants (ref: White)**

|  | 3-month | | | | | |
| --- | --- | --- | --- | --- | --- | --- |
| Race Group | Asian | | Black | | Other/Multiple | |
| Symptom category | Estimated **adjusted** OR (95% CI) | p-value | Estimated **adjusted** OR (95% CI) | p-value | Estimated **adjusted** OR (95% CI) | p-value |
| Constitutional |  |  |  |  |  |  |
| Tired | 0.81 (0.55, 1.21) | 0.31 | 0.78 (0.51, 1.19) | 0.25 | 1.34 (0.94, 1.91) | 0.10 |
| Chills | 0.92 (0.50, 1.73) | 0.81 | 0.80 (0.42, 1.52) | 0.49 | 1.23 (0.72, 2.12) | 0.45 |
| Feeling hot | 1.07 (0.56, 2.04) | 0.85 | 0.96 (0.50, 1.85) | 0.90 | 1.44 (0.83, 2.52) | 0.20 |
| Fever | 0.92 (0.38, 2.23) | 0.85 | 0.63 (0.24, 1.65) | 0.35 | 1.22 (0.58, 2.57) | 0.60 |
| Shakes | 1.18 (0.41, 3.33) | 0.76 | 0.75 (0.29, 1.95) | 0.55 | 0.78 (0.26, 2.30) | 0.65 |
| HEENT^+^ |  |  |  |  |  |  |
| Headache | 0.77 (0.48, 1.23) | 0.27 | 0.89 (0.55, 1.44) | 0.64 | 1.38 (0.93, 2.04) | 0.11 |
| Runny nose | 1.00 (0.62, 1.62) | 0.99 | 0.75 (0.40, 1.38) | 0.36 | 1.35 (0.86, 2.13) | 0.20 |
| Loss of smell | 0.90 (0.57, 1.44) | 0.67 | 0.45 (0.25, 0.82) | <.01^**^ | 0.72 (0.44, 1.16) | 0.17 |
| Loss of taste | 1.00 (0.61, 1.64) | 0.99 | 0.68 (0.39, 1.18) | 0.17 | 0.90 (0.54, 1.48) | 0.67 |
| Sore throat | 1.72 (1.09, 2.72) | 0.02^*^ | 1.08 (0.59, 1.96) | 0.80 | 1.44 (0.87, 2.37) | 0.15 |
| Loss of hair | 0.84 (0.40, 1.78) | 0.64 | 0.78 (0.39, 1.55) | 0.47 | 0.97 (0.49, 1.93) | 0.93 |
| Pulmonary |  |  |  |  |  |  |
| Cough | 1.57 (0.96, 2.58) | 0.07 | 1.19 (0.66, 2.14) | 0.56 | 1.43 (0.85, 2.40) | 0.18 |
| Shortness of breath | 0.32 (0.14, 0.74) | <.01^**^ | 0.55 (0.30, 1.01) | 0.05 | 1.28 (0.80, 2.06) | 0.31 |
| Wheezing | 1.22 (0.41, 3.67) | 0.72 | 0.29 (0.09, 0.98) | 0.05^*^ | 2.22 (1.04, 4.75) | 0.04^*^ |
| Cardiovascular | 0.73 (0.38, 1.42) | 0.36 | 0.95 (0.54, 1.68) | 0.87 | 1.09 (0.63, 1.91) | 0.76 |
| Chest pains | 0.80 (0.36, 1.76) | 0.58 | 0.67 (0.32, 1.42) | 0.30 | 0.92 (0.47, 1.83) | 0.82 |
| Palpitations | 0.65 (0.25, 1.66) | 0.37 | 0.86 (0.39, 1.94) | 0.72 | 1.04 (0.46, 2.32) | 0.93 |
| Gastrointestinal |  |  |  |  |  |  |
| Diarrhea | 0.56 (0.21, 1.53) | 0.26 | 0.46 (0.19, 1.09) | 0.08 | 0.90 (0.43, 1.87) | 0.78 |
| Nausea or vomiting | 0.90 (0.33, 2.45) | 0.83 | 0.64 (0.27, 1.51) | 0.31 | 1.36 (0.65, 2.85) | 0.42 |
| Abdominal pain | 0.55 (0.16, 1.95) | 0.35 | 0.47 (0.17, 1.28) | 0.14 | 1.34 (0.62, 2.88) | 0.46 |
| Musculoskeletal |  |  |  |  |  |  |
| Aches | 0.94 (0.59, 1.48) | 0.78 | 0.80 (0.48, 1.34) | 0.40 | 1.32 (0.85, 2.03) | 0.21 |
| Joint pains | 0.99 (0.59, 1.69) | 0.98 | 1.03 (0.63, 1.68) | 0.91 | 1.21 (0.75, 1.95) | 0.44 |
| Symptom summary |  |  |  |  |  |  |
| Other symptoms | 0.37 (0.13, 1.05) | 0.06 | 0.35 (0.13, 0.95) | 0.04^*^ | 1.38 (0.72, 2.66) | 0.33 |
| ≥3 symptoms (not including other) | 0.96 (0.64, 1.42) | 0.82 | 0.73 (0.47, 1.14) | 0.17 | 1.27 (0.89, 1.83) | 0.19 |
| No symptoms | 1.16 (0.83, 1.61) | 0.39 | 1.32 (0.91, 1.92) | 0.14 | 0.85 (0.62, 1.18) | 0.34 |
| Activity Level (ref: same as before) |  |  |  |  |  |  |
| Somewhat less than before | 0.86 (0.58, 1.28) | 0.46 | 0.82 (0.53, 1.28) | 0.39 | 1.72 (1.21, 2.46) | <.01^**^ |
| Much less than before | 0.42 (0.15, 1.16) | 0.09 | 0.87 (0.48, 1.56) | 0.63 | 2.08 (1.18, 3.65) | 0.01^*^ |
| Missed work due to health reasons past 3 months (ref: 0-5 workdays) |  |  |  |  |  |  |
| Missed >5 workdays | 0.98 (0.60, 1.60) | 0.92 | 1.57 (1.00, 2.48) | 0.05 | 1.32 (0.86, 2.04) | 0.21 |
| Health status (ref: good) |  | |  |  |  |  |
| Excellent/Very good | 0.69 (0.47, 1.01) | 0.06 | 1.24 (0.75, 2.05) | 0.40 | 1.03 (0.67, 1.57) | 0.91 |
| Fair/Poor | 1.38 (0.91, 2.10) | 0.13 | 0.91 (0.58, 1.42) | 0.68 | 1.90 (1.25, 2.88) | <.01^**^ |

^*^p-value <.05; ^**^p-value < .01; ^+^Head, ears, eyes, nose, throat

**Supplementary Material 5.2b. GEE adjusted OR output for race at 3-month among SARS-CoV-2-negative INSPIRE participants (ref: White)**

|  | 3-month | | | | | |
| --- | --- | --- | --- | --- | --- | --- |
| Race Group | Asian | | Black | | Other/Multiple | |
| Symptom category | Estimated **adjusted** OR (95% CI) | p-value | Estimated **adjusted** OR (95% CI) | p-value | Estimated **adjusted** OR (95% CI) | p-value |
| Constitutional |  |  |  |  |  |  |
| Tired | 0.62 (0.35, 1.08) | 0.09 | 0.70 (0.41, 1.21) | 0.2 | 1.27 (0.71, 2.27) | 0.42 |
| Chills | 0.70 (0.33, 1.47) | 0.35 | 0.52 (0.23, 1.18) | 0.12 | 0.97 (0.41, 2.33) | 0.95 |
| Feeling hot | 1.09 (0.51, 2.32) | 0.83 | 0.43 (0.18, 1.05) | 0.06 | 0.67 (0.24, 1.82) | 0.43 |
| Fever | 0.75 (0.28, 2.02) | 0.57 | 0.40 (0.13, 1.19) | 0.1 | 0.85 (0.33, 2.21) | 0.74 |
| Shakes | 1.49 (0.49, 4.54) | 0.48 | 0.83 (0.24, 2.86) | 0.77 | 0.68 (0.16, 2.96) | 0.61 |
| HEENT^+^ |  |  |  |  |  |  |
| Headache | 0.65 (0.38, 1.13) | 0.13 | 0.62 (0.33, 1.13) | 0.12 | 1.06 (0.59, 1.91) | 0.84 |
| Runny nose | 0.77 (0.41, 1.45) | 0.42 | 0.63 (0.33, 1.20) | 0.16 | 1.11 (0.56, 2.19) | 0.77 |
| Loss of smell | 1.24 (0.55, 2.81) | 0.6 | 0.38 (0.10, 1.49) | 0.16 | 0.34 (0.08, 1.46) | 0.15 |
| Loss of taste | 2.55 (1.10, 5.94) | 0.03* | 0.58 (0.14, 2.43) | 0.46 | 0.67 (0.19, 2.37) | 0.54 |
| Sore throat | 0.79 (0.41, 1.52) | 0.47 | 0.60 (0.30, 1.17) | 0.13 | 1.34 (0.72, 2.48) | 0.36 |
| Loss of hair | 0.79 (0.22, 2.90) | 0.73 | 1.47 (0.51, 4.28) | 0.48 | 0.26 (0.03, 1.97) | 0.19 |
| Pulmonary |  |  |  |  |  |  |
| Cough | 1.09 (0.56, 2.11) | 0.81 | 0.86 (0.40, 1.83) | 0.69 | 1.20 (0.58, 2.48) | 0.62 |
| Shortness of breath | 0.52 (0.23, 1.16) | 0.11 | 0.30 (0.11, 0.84) | 0.02* | 0.96 (0.42, 2.20) | 0.93 |
| Wheezing | 0.35 (0.08, 1.54) | 0.16 | 0.46 (0.09, 2.37) | 0.35 | 0.60 (0.11, 3.31) | 0.56 |
| Cardiovascular | 0.68 (0.31, 1.51) | 0.34 | 0.78 (0.35, 1.73) | 0.54 | 0.92 (0.42, 2.02) | 0.84 |
| Chest pains | 0.49 (0.20, 1.21) | 0.12 | 0.63 (0.24, 1.67) | 0.35 | 0.72 (0.25, 2.06) | 0.54 |
| Palpitations | 1.04 (0.36, 2.96) | 0.94 | 1.18 (0.40, 3.49) | 0.76 | 1.07 (0.41, 2.79) | 0.89 |
| Gastrointestinal |  |  |  |  |  |  |
| Diarrhea | 0.68 (0.25, 1.86) | 0.45 | 0.60 (0.22, 1.59) | 0.3 | 1.22 (0.47, 3.15) | 0.68 |
| Nausea or vomiting | 1.03 (0.43, 2.51) | 0.94 | 0.33 (0.11, 0.96) | 0.04* | 0.60 (0.17, 2.03) | 0.41 |
| Abdominal pain | 1.15 (0.41, 3.28) | 0.79 | 0.52 (0.13, 2.11) | 0.36 | 1.71 (0.53, 5.49) | 0.36 |
| Musculoskeletal |  |  |  |  |  |  |
| Aches | 0.85 (0.48, 1.50) | 0.57 | 0.68 (0.35, 1.32) | 0.26 | 1.12 (0.58, 2.16) | 0.74 |
| Joint pains | 0.94 (0.48, 1.83) | 0.85 | 0.64 (0.28, 1.44) | 0.28 | 1.39 (0.69, 2.80) | 0.35 |
| Symptom summary |  |  |  |  |  |  |
| Other symptoms | 0.41 (0.10, 1.73) | 0.22 | 0.74 (0.25, 2.23) | 0.60 | 0.77 (0.25, 2.40) | 0.65 |
| ≥3 symptoms (not including other) | 0.72 (0.43, 1.20) | 0.20 | 0.64 (0.34, 1.20) | 0.17 | 1.25 (0.70, 2.25) | 0.45 |
| No symptoms | 1.52 (0.90, 2.57) | 0.12 | 1.51 (0.89, 2.54) | 0.13 | 0.82 (0.49, 1.37) | 0.44 |
| Activity level (ref: same as before) |  |  |  |  |  |  |
| Somewhat less than before | 0.39 (0.17, 0.89) | 0.03* | 1.36 (0.69, 2.66) | 0.38 | 0.88 (0.38, 2.02) | 0.76 |
| Much less than before | 0.42 (0.17, 1.05) | 0.06 | 0.93 (0.29, 3.02) | 0.91 | 0.92 (0.31, 2.76) | 0.88 |
| Missed work due to health reasons past 3 months (ref: 0-5 workdays) |  |  |  |  |  |  |
| Missed >5 workdays | 2.67 (1.18, 6.07) | 0.02* | 0.93 (0.34, 2.53) | 0.89 | 2.11 (0.78, 5.71) | 0.14 |
| Health status (ref: good) |  | |  |  |  |  |
| Excellent/Very good | 2.13 (1.04, 4.38) | 0.04* | 0.81 (0.41, 1.63) | 0.56 | 1.04 (0.46, 2.36) | 0.93 |
| Fair/Poor | 0.45 (0.22, 0.93) | 0.03* | 1.14 (0.56, 2.32) | 0.72 | 1.39 (0.64, 3.01) | 0.41 |

^*^p-value <.05; ^**^p-value < .01; ^+^Head, ears, eyes, nose, throat

**Supplementary Material 5.3a. GEE adjusted OR output for race at 6-month among SARS-CoV-2-positive INSPIRE participants (ref: White)**

|  | 6-month | | | | | |
| --- | --- | --- | --- | --- | --- | --- |
| Race group | Asian | | Black | | Other/Multiple | |
| Symptom category | Estimated **adjusted** OR (95% CI) | p-value | Estimated **adjusted** OR (95% CI) | p-value | Estimated **adjusted** OR (95% CI) | p-value |
| Constitutional |  |  |  |  |  |  |
| Tired | 0.97 (0.55, 1.69) | 0.91 | 1.12 (0.67, 1.89) | 0.66 | 1.08 (0.63, 1.87) | 0.78 |
| Chills | 1.66 (0.80, 3.43) | 0.17 | 0.92 (0.39, 2.17) | 0.85 | 0.62 (0.20, 1.96) | 0.42 |
| Feeling hot | 1.35 (0.60, 3.03) | 0.47 | 0.74 (0.30, 1.85) | 0.52 | 0.84 (0.32, 2.22) | 0.72 |
| Fever | 2.09 (0.69, 6.40) | 0.19 | 0.73 (0.15, 3.55) | 0.70 | 1.53 (0.46, 5.13) | 0.49 |
| Shakes | 2.46 (0.71, 8.54) | 0.16 | 0.38 (0.06, 2.65) | 0.33 | 0.49 (0.05, 5.08) | 0.55 |
| HEENT^+^ |  |  |  |  |  |  |
| Headache | 0.91 (0.49, 1.67) | 0.76 | 1.01 (0.56, 1.85) | 0.96 | 1.29 (0.72, 2.31) | 0.38 |
| Runny nose | 1.15 (0.63, 2.09) | 0.65 | 1.10 (0.57, 2.14) | 0.77 | 1.03 (0.53, 2.00) | 0.92 |
| Loss of smell | 1.09 (0.53, 2.24) | 0.81 | 0.76 (0.37, 1.58) | 0.46 | 0.39 (0.14, 1.12) | 0.08 |
| Loss of taste | 0.92 (0.41, 2.05) | 0.83 | 0.75 (0.35, 1.61) | 0.46 | 0.23 (0.05, 0.95) | 0.04^*^ |
| Sore throat | 1.11 (0.56, 2.22) | 0.76 | 0.97 (0.44, 2.18) | 0.95 | 1.13 (0.56, 2.29) | 0.73 |
| Loss of hair | 1.04 (0.45, 2.40) | 0.93 | 0.58 (0.22, 1.55) | 0.28 | 1.24 (0.57, 2.69) | 0.58 |
| Pulmonary |  |  |  |  |  |  |
| Cough | 1.30 (0.62, 2.75) | 0.49 | 1.13 (0.50, 2.55) | 0.78 | 1.04 (0.47, 2.34) | 0.92 |
| Shortness of breath | 0.51 (0.18, 1.39) | 0.19 | 0.83 (0.41, 1.65) | 0.59 | 1.34 (0.71, 2.56) | 0.37 |
| Wheezing | 0.54 (0.11, 2.75) | 0.46 | 0.57 (0.17, 1.90) | 0.36 | 1.85 (0.67, 5.15) | 0.24 |
| Cardiovascular |  |  |  |  |  |  |
| Chest pains | 1.18 (0.45, 3.10) | 0.73 | 0.75 (0.28, 1.99) | 0.56 | 0.91 (0.34, 2.44) | 0.85 |
| Palpitations | 0.34 (0.06, 1.78) | 0.20 | 0.84 (0.31, 2.26) | 0.73 | 1.58 (0.66, 3.80) | 0.31 |
| Gastrointestinal |  |  |  |  |  |  |
| Diarrhea | 1.42 (0.52, 3.83) | 0.49 | 0.86 (0.29, 2.53) | 0.78 | 1.06 (0.35, 3.16) | 0.92 |
| Nausea or vomiting | 1.09 (0.36, 3.27) | 0.88 | 1.02 (0.38, 2.72) | 0.98 | 1.00 (0.34, 2.99) | 0.99 |
| Abdominal pain | 0.78 (0.28, 2.22) | 0.64 | 0.44 (0.13, 1.48) | 0.18 | 0.87 (0.29, 2.60) | 0.80 |
| Musculoskeletal |  |  |  |  |  |  |
| Aches | 1.17 (0.66, 2.06) | 0.59 | 1.17 (0.67, 2.05) | 0.58 | 0.96 (0.52, 1.78) | 0.89 |
| Joint pains | 0.48 (0.22, 1.03) | 0.06 | 0.79 (0.41, 1.53) | 0.49 | 0.90 (0.47, 1.73) | 0.75 |
| Symptom summary |  |  |  |  |  |  |
| Other symptoms | 0.75 (0.27, 2.09) | 0.58 | 0.37 (0.09, 1.59) | 0.18 | 1.25 (0.50, 3.16) | 0.63 |
| ≥3 symptoms (not including other) | 0.99 (0.59, 1.69) | 0.98 | 0.91 (0.55, 1.51) | 0.71 | 1.14 (0.69, 1.89) | 0.61 |
| No symptoms | 0.87 (0.58, 1.30) | 0.49 | 1.21 (0.78, 1.89) | 0.39 | 0.75 (0.50, 1.14) | 0.18 |
| Activity level (ref: same as before) |  |  |  |  |  |  |
| Somewhat less than before | 1.06 (0.67, 1.67) | 0.82 | 0.71 (0.41, 1.23) | 0.22 | 1.60 (1.02, 2.51) | 0.04^*^ |
| Much less than before | 0.57 (0.23, 1.42) | 0.23 | 0.71 (0.35, 1.43) | 0.33 | 2.49 (1.40, 4.44) | <.01^**^ |
| Missed work due to health reasons past 3 months (ref: 0-5 workdays) |  |  |  |  |  |  |
| Missed >5 workdays | 1.29 (0.66, 2.52) | 0.46 | 2.83 (1.60, 5.00) | <.01^**^ | 2.25 (1.27, 3.98) | <.01^**^ |
| Health status (ref: good) |  |  |  |  |  |  |
| Excellent/Very good | 1.04 (0.66, 1.64) | 0.87 | 1.28 (0.71, 2.31) | 0.42 | 1.30 (0.77, 2.19) | 0.32 |
| Fair/Poor | 1.88 (1.13, 3.12) | 0.02^*^ | 0.75 (0.43, 1.29) | 0.30 | 1.83 (1.10, 3.05) | 0.02^*^ |

^*^p-value <.05; ^**^p-value < .01; ^+^Head, ears, eyes, nose, throat

**eTable 5.3b: GEE adjusted OR output for race at 6-month among SARS-CoV-2-negative INSPIRE participants (ref: White)**

|  | 6-month | | | | | |
| --- | --- | --- | --- | --- | --- | --- |
| Race group | Asian | | Black | | Other/Multiple | |
| Symptom category | Estimated **adjusted** OR (95% CI) | p-value | Estimated **adjusted** OR (95% CI) | p-value | Estimated **adjusted** OR (95% CI) | p-value |
| Constitutional |  |  |  |  |  |  |
| Tired | 0.65 (0.33, 1.28) | 0.21 | 0.97 (0.52, 1.82) | 0.92 | 1.17 (0.63, 2.16) | 0.62 |
| Chills | 0.81 (0.30, 2.18) | 0.67 | 0.94 (0.38, 2.31) | 0.89 | 0.49 (0.14, 1.71) | 0.26 |
| Feeling hot | 0.84 (0.29, 2.40) | 0.74 | 0.55 (0.21, 1.41) | 0.21 | 0.39 (0.10, 1.51) | 0.17 |
| Fever | 0.87 (0.17, 4.48) | 0.87 | 0.91 (0.27, 3.08) | 0.88 | 1.07 (0.26, 4.41) | 0.93 |
| Shakes | 0.77 (0.10, 5.78) | 0.8 | 1.74 (0.43, 7.04) | 0.44 | 0.43 (0.04, 4.59) | 0.49 |
| HEENT^+^ |  |  |  |  |  |  |
| Headache | 0.74 (0.37, 1.48) | 0.39 | 0.73 (0.37, 1.46) | 0.37 | 1.00 (0.49, 2.03) | 0.99 |
| Runny nose | 1.14 (0.53, 2.44) | 0.74 | 0.72 (0.35, 1.49) | 0.38 | 0.85 (0.38, 1.89) | 0.69 |
| Loss of smell | 2.10 (0.81, 5.43) | 0.13 | 0.46 (0.11, 1.95) | 0.29 | 0.19 (0.03, 1.05) | 0.06 |
| Loss of taste | 2.81 (1.07, 7.40) | 0.04* | 0.53 (0.10, 2.90) | 0.47 | 0.17 (0.03, 1.09) | 0.06 |
| Sore throat | 0.71 (0.30, 1.67) | 0.43 | 0.38 (0.16, 0.93) | 0.03* | 1.05 (0.47, 2.36) | 0.9 |
| Loss of hair | 0.59 (0.14, 2.62) | 0.49 | 1.83 (0.56, 5.94) | 0.32 | 0.33 (0.04, 2.60) | 0.29 |
| Pulmonary |  |  |  |  |  |  |
| Cough | 1.03 (0.40, 2.64) | 0.95 | 0.71 (0.29, 1.77) | 0.46 | 0.88 (0.33, 2.32) | 0.79 |
| Shortness of breath | 0.78 (0.31, 1.97) | 0.6 | 0.47 (0.16, 1.40) | 0.18 | 1.01 (0.42, 2.43) | 0.98 |
| Wheezing | 0.67 (0.16, 2.83) | 0.59 | 0.20 (0.03, 1.34) | 0.1 | 0.50 (0.10, 2.58) | 0.41 |
| Cardiovascular | 0.66 (0.24, 1.84) | 0.43 | 1.10 (0.45, 2.70) | 0.83 | 1.23 (0.50, 3.01) | 0.65 |
| Chest pains | 0.55 (0.16, 1.88) | 0.34 | 0.93 (0.34, 2.58) | 0.89 | 0.71 (0.22, 2.25) | 0.56 |
| Palpitations | 1.01 (0.31, 3.32) | 0.98 | 0.61 (0.11, 3.38) | 0.57 | 1.63 (0.50, 5.29) | 0.42 |
| Gastrointestinal |  |  |  |  |  |  |
| Diarrhea | 1.26 (0.39, 4.11) | 0.7 | 1.50 (0.50, 4.51) | 0.47 | 1.43 (0.44, 4.68) | 0.55 |
| Nausea or vomiting | 1.64 (0.56, 4.80) | 0.37 | 0.40 (0.08, 1.93) | 0.25 | 0.44 (0.11, 1.78) | 0.25 |
| Abdominal pain | 1.07 (0.29, 4.02) | 0.92 | 0.74 (0.12, 4.47) | 0.75 | 1.11 (0.32, 3.81) | 0.86 |
| Musculoskeletal |  |  |  |  |  |  |
| Aches | 1.24 (0.60, 2.55) | 0.56 | 0.85 (0.41, 1.77) | 0.67 | 0.81 (0.36, 1.83) | 0.62 |
| Joint pains | 0.72 (0.31, 1.67) | 0.45 | 0.31 (0.10, 0.94) | 0.04* | 1.03 (0.45, 2.38) | 0.94 |
| Symptom summary |  |  |  |  |  |  |
| Other symptoms | 0.44 (0.09, 2.19) | 0.31 | 1.48 (0.41, 5.37) | 0.55 | 0.70 (0.18, 2.72) | 0.60 |
| ≥3 symptoms (not including other) | 0.89 (0.47, 1.69) | 0.72 | 0.67 (0.33, 1.35) | 0.26 | 1.12 (0.57, 2.20) | 0.73 |
| No symptoms | 1.39 (0.75, 2.56) | 0.29 | 1.13 (0.64, 2.00) | 0.68 | 0.72 (0.42, 1.25) | 0.24 |
| Activity level (ref: same as before) |  |  |  |  |  |  |
| Somewhat less than before | 0.45 (0.22, 0.93) | 0.03* | 1.10 (0.59, 2.07) | 0.76 | 0.95 (0.43, 2.08) | 0.90 |
| Much less than before | 0.52 (0.22, 1.23) | 0.14 | 0.68 (0.27, 1.69) | 0.41 | 0.76 (0.26, 2.27) | 0.63 |
| Missed work missed due to health reasons past 3 months (ref: 0-5 workdays) |  |  |  |  |  |  |
| Missed >5 workdays | 1.49 (0.73, 3.03) | 0.28 | 0.71 (0.28, 1.75) | 0.45 | 1.24 (0.49, 3.14) | 0.65 |
| Health status (ref: good) |  |  |  |  |  |  |
| Excellent/Very good | 2.07 (1.10, 3.90) | 0.02* | 0.54 (0.28, 1.04) | 0.06 | 0.82 (0.36, 1.89) | 0.64 |
| Fair/Poor | 0.55 (0.31, 0.99) | 0.05 | 0.84 (0.46, 1.51) | 0.55 | 1.44 (0.71, 2.90) | 0.31 |

^*^p-value <.05; ^**^p-value < .01; ^+^Head, ears, eyes, nose, throa

**
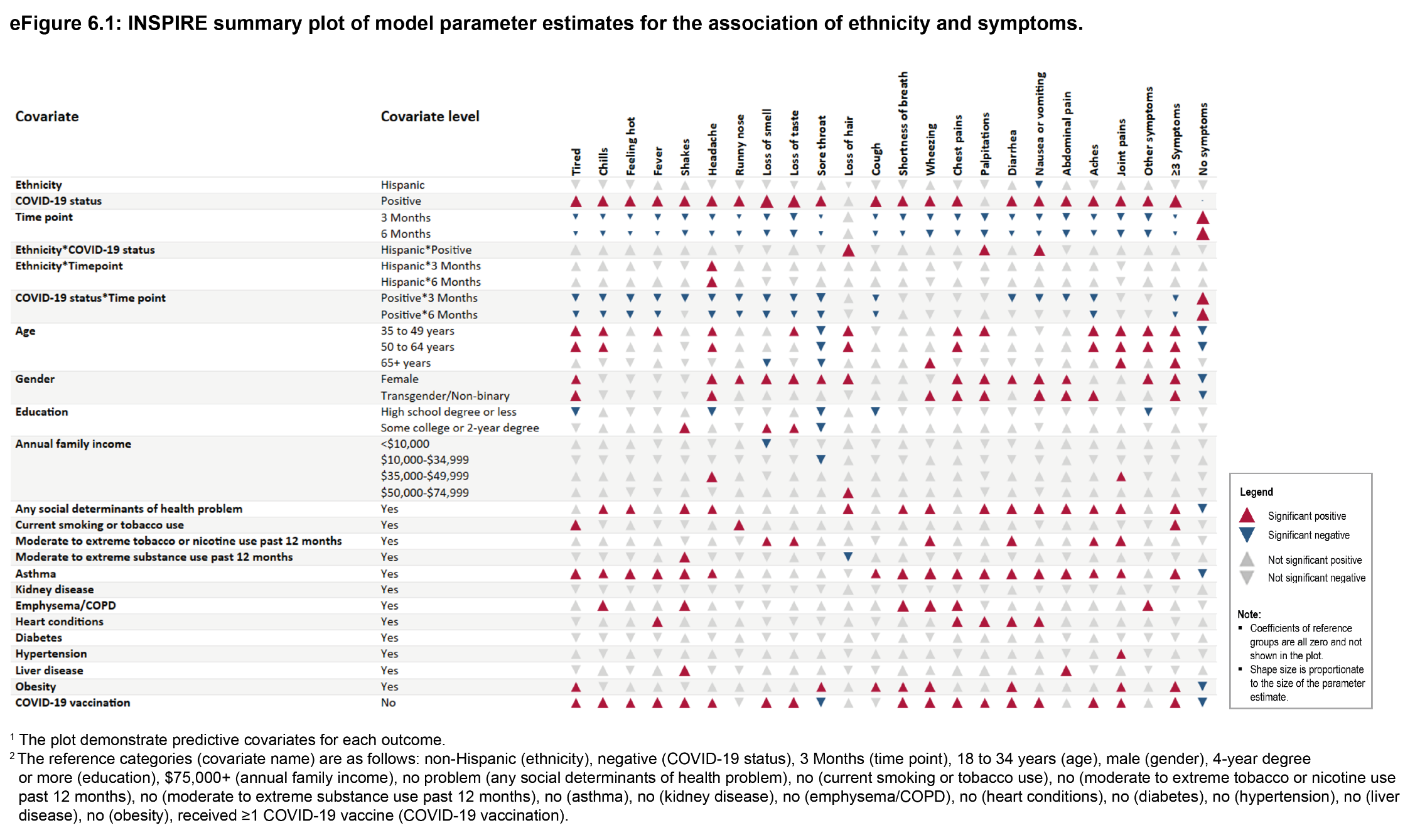
**


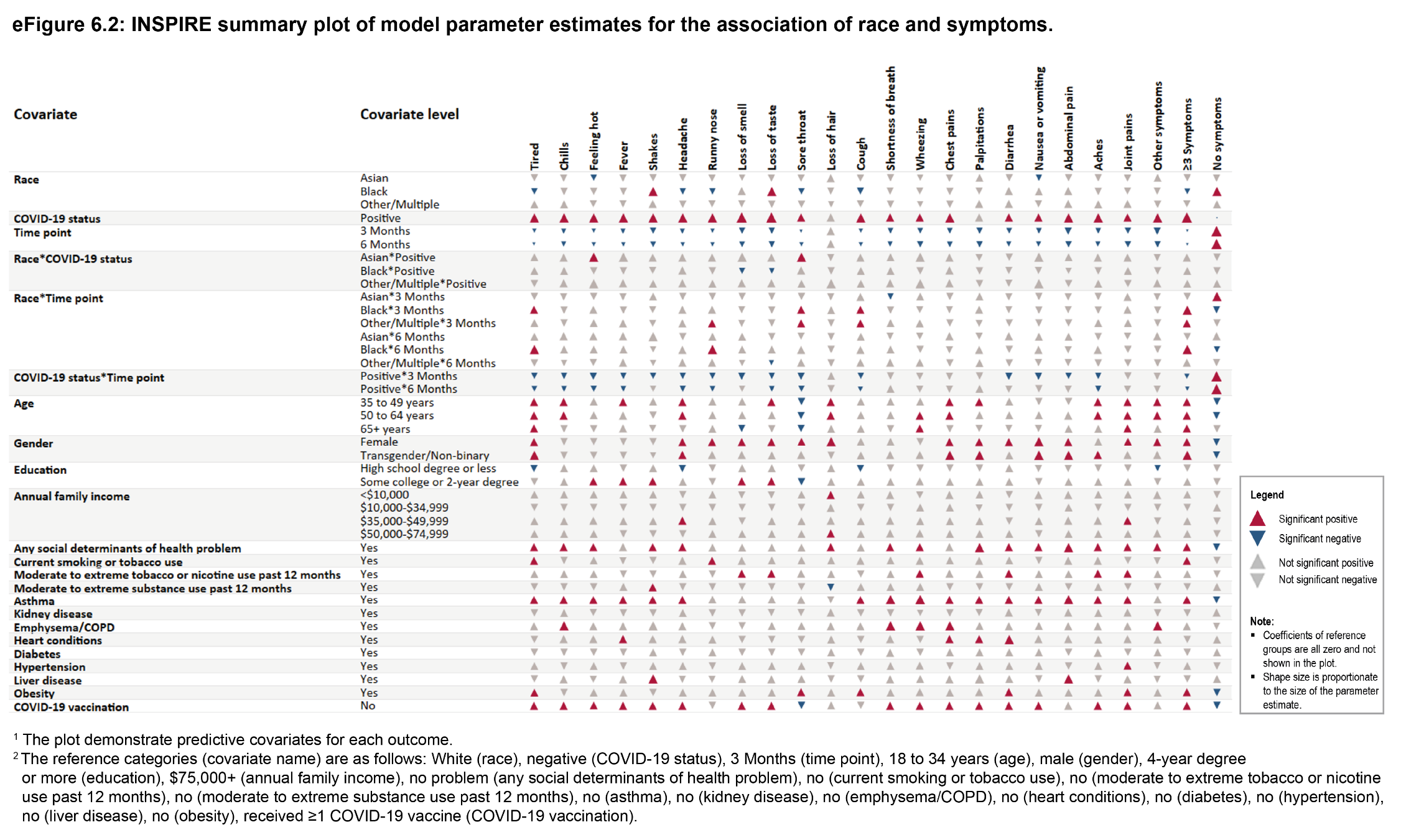


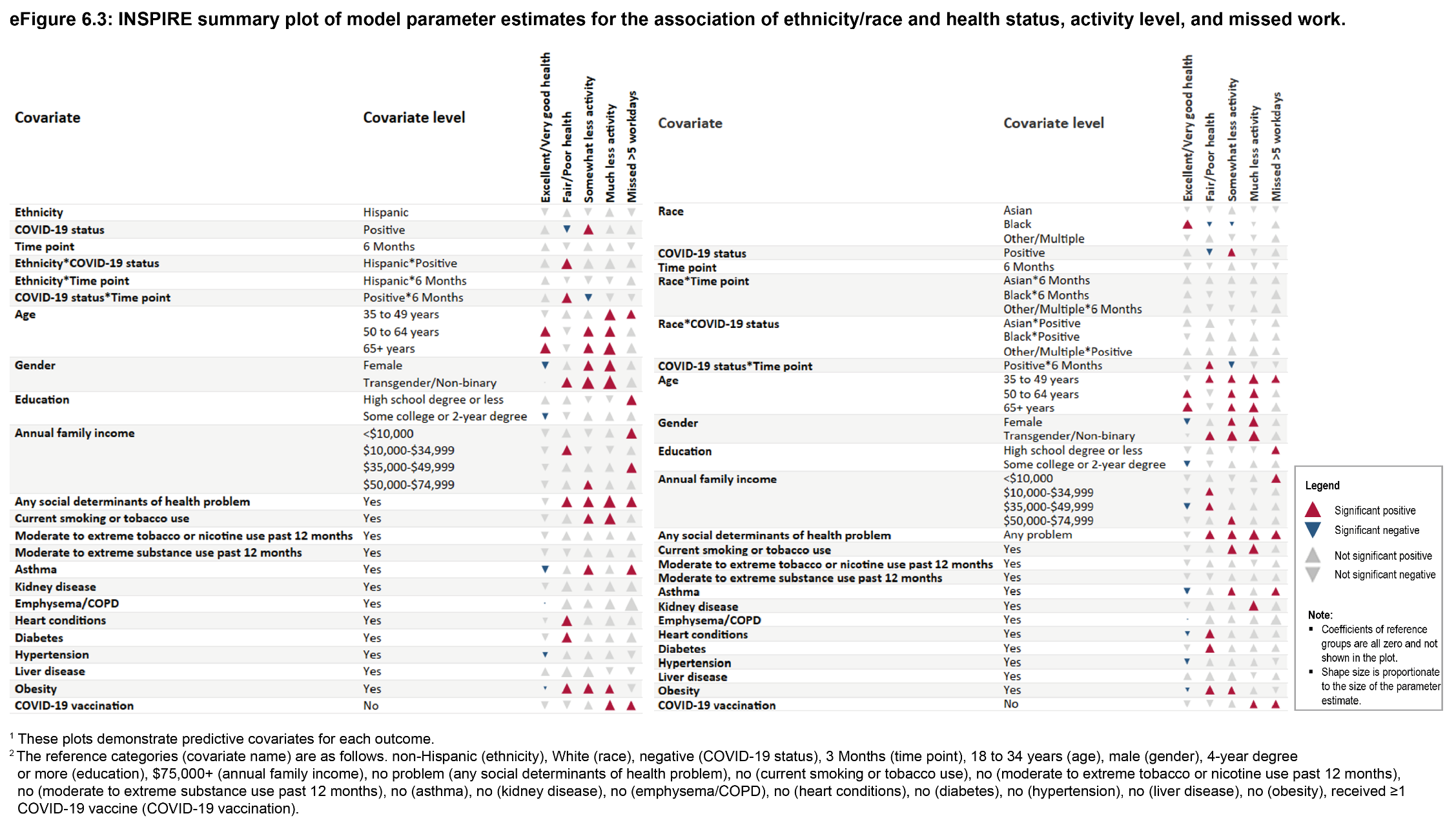

Supplement: Supplementary file 1 [file Table_1.docx]
